# Structural features of the interaction of MapZ with FtsZ and membranes in *Streptococcus pneumoniae*

**DOI:** 10.1038/s41598-020-61036-9

**Published:** 2020-03-04

**Authors:** Tomas Hosek, Catherine M. Bougault, Jean-Pierre Lavergne, Denis Martinez, Isabel Ayala, Daphna Fenel, Marine Restelli, Cecile Morlot, Birgit Habenstein, Christophe Grangeasse, Jean-Pierre Simorre

**Affiliations:** 1 Univ. Grenoble Alpes, CNRS, CEA, Institut de Biologie Structurale, F-38000 Grenoble, France; 2 Molecular Microbiology and Structural Biochemistry, CNRS UMR 5086, Université de Lyon, Lyon, France; 3 Institute of Chemistry and Biology of Membranes and Nano-objects, CBMN-CNRS Université de Bordeaux, Pessac, France

**Keywords:** Bacterial development, Bacterial structural biology, Pathogens, Cell division, Structural biology, Electron microscopy, NMR spectroscopy

## Abstract

MapZ localizes at midcell and acts as a molecular beacon for the positioning of the cell division machinery in the bacterium *Streptococcus pneumoniae*. MapZ contains a single transmembrane helix that separates the C-terminal extracellular domain from the N-terminal cytoplasmic domain. Only the structure and function of the extracellular domain is known. Here, we demonstrate that large parts of the cytoplasmic domain is intrinsically disordered and that there are two regions (from residues 45 to 68 and 79 to 95) with a tendency to fold into amphipathic helices. We further reveal that these regions interact with the surface of liposomes that mimic the *Streptococcus pneumoniae* cell membrane. The highly conserved and unfolded N-terminal region (from residues 17 to 43) specifically interacts with FtsZ independently of FtsZ polymerization state. Moreover, we show that MapZ phosphorylation at positions Thr67 and Thr68 does not impact the interaction with FtsZ or liposomes. Altogether, we propose a model in which the MapZ-mediated recruitment of FtsZ to mid-cell is modulated through competition of MapZ binding to the cell membrane. The molecular interplay between the components of this tripartite complex could represent a key step toward the complete assembly of the divisome.

## Introduction

The initiation of bacterial cell division requires the identification of the cell’s midpoint prior to the assembly of a highly dynamic protein complex, called the divisome^1^. The divisome constricts the cell and assembles the cell wall depending on the tubulin-like protein FtsZ^2^. FtsZ polymerizes into protofilaments that ultimately assemble as bundles or clusters, forming a highly dynamic ring called the Z-ring^3^. Treadmilling of FtsZ filaments, a process that is fine-tuned by several accessory proteins, allows for cell constriction together with the assembly of the septal cell wall, giving rise to the two newborn cells. Therefore, a key event for successful division of the bacterial cell is the selection of the site of cell division and the proper positioning of FtsZ.

The positioning of the Z-ring at the cell center should not interfere with the replication and segregation of the chromosome. In *Escherichia coli* and *Bacillus subtilis*, the nucleoid occlusion system (NO)^4^ and the Min system^5^ allows the assembly of the Z-ring at mid-cell only when chromosome replication and segregation are completed^6^. However, some bacteria use other mechanisms to position the Z-ring at the cell center. For instance, Z-ring positioning is governed by the protein MipZ in the bacterium *Caulobacter crescentus*^7^. This protein is conserved in gamma-proteobacteria and also prevents the assembly of Z-ring near the cell poles. By contrast, bacteria like *Streptomyces coelicolor*^8^ and *Myxococcus xanthus*^9^ employ alternative mechanisms unrelated to the NO, Min, and MipZ systems. SsgAB in *S. coelicolor* and PomZ in *M. xanthus* localize to the mid-cell prior to FtsZ, where they promote FtsZ polymerization and positively drive the assembly of the Z-ring. However, it still remains unclear how these two systems localize at mid-cell.

In the human pathogen *Streptococus pneumoniae*, the mid-cell positioning of FtsZ is proposed to rely on chromosome segregation^10^ and the MapZ protein (alternately termed LocZ)^11,12^. *S. pneumoniae* is an ovoid-shaped bacterium that maintains its characteristic cell shape by the coordinated peptidoglycan (PG) assembly at mid-cell, producing the new cell-hemispheres in between the old hemispheres^13^. Consequently, the boundary between new and old cell wall material (*i.e*. the cell equator) moves away from mid-cell as the cell elongates^14^. At the beginning of the cell cycle, MapZ forms a stable ring structure at mid-cell, which is proposed to act as an anchor to guide FtsZ treadmilling^11,15^. Just before constriction begins, the MapZ ring splits into two rings which move apart during cell growth to eventually mark the future cell division sites of the two daughter cells. MapZ is a membrane protein with a single transmembrane helix separating a cytoplasmic amino-terminal domain and an extracellular carboxy-terminal domain. The structure of the extracellular domain of MapZ shows a bi-modular organization with two structured subdomains separated by a flexible linker^16^. The membrane proximal-domain serves as a pedestal for the membrane distal C-terminal domain, which interacts with PG. The interaction with PG allows the two MapZ rings to separate at the speed of cell elongation. The extracellular domain of MapZ thus allows the protein to position at the future division sites, while the N-terminal domain guides FtsZ positioning through direct interaction. Little information is available concerning the structural organization of the cytoplasmic domain of MapZ and its interaction with FtsZ^15,17^. It was reported that the N-terminal region of MapZ, from residue 1 to 41, is required for the direct interaction with FtsZ^11^. In addition, the cytoplasmic domain of MapZ can be phosphorylated on two distinct threonine residues (T67 and T78) by StkP, a serine/threonine protein-kinase crucial for cell division and morphogenesis*s*^18^. While MapZ phosphorylation seems to be crucial for cell constriction, it has no impact on the positioning of FtsZ at mid-cell, suggesting that MapZ could be involved in cell division at two temporally distinct steps.

To gain structural knowledge about the cytoplasmic domain of MapZ and to decipher the details of its interaction with FtsZ, we used several Nuclear Magnetic Resonance (NMR) spectroscopy approaches. We show that the domain is intrinsically highly disordered. We demonstrate that the N-terminal domain of MapZ interacts with FtsZ monomers and protofilaments as well as with negatively charged lipids, suggesting that this region also interacts with the cell membrane. We further provide evidence that the acyl chain of lipids are more mobile in the presence of MapZ_*cyto*_, suggesting that the interaction between MapZ and the membrane influences the recruitment of FtsZ and the assembly of the Z-ring at mid-cell. Last, we found that phosphorylation of MapZ_*cyto*_ does not significantly affect these interactions. Altogether, this work provides detailed structural insights toward the ultimate understanding of the role of the MapZ in the cell division of *S. pneumoniae*.

## Results and Discussion

### MapZ intracellular domain is intrinsically disordered

**Figure 1:**
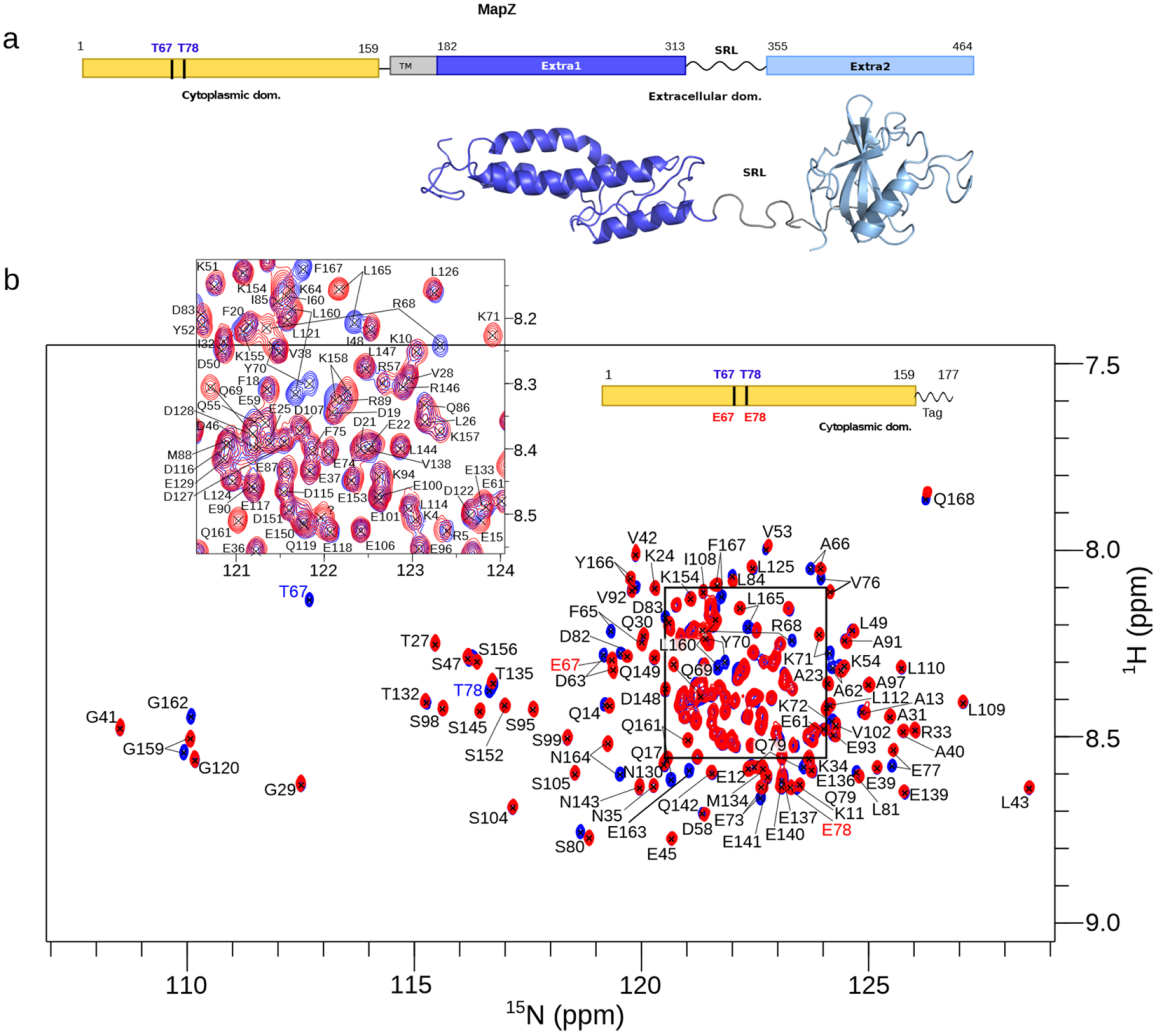
Structural features of MapZ from *S. pneumoniae*. (**a**) Domain organization of the full-length MapZ protein of *S. pneumoniae*. The protein consists of a cytoplasmic domain of 159 residues (yellow), linked by a transmembrane helix (grey) to an extracellular domain of 282 residues. Tertiary structure of this extra-cellular domain has been solved^16^ and reveals two structurally independent domains (dark and light blue) linked by a flexible Serine-Rich Linker (SRL). (**b**) Overlay of the ^1^H–^15^N BEST-TROSY spectra recorded for ^13^C,^15^N-labeled wild-type MapZ_*cyto*_ (blue) and MapZ_*cyto*_^2*TE*^ phosphomimetic construct (T67E/T78E MapZ_*cyto*_) (red). Protein samples were prepared at 0.2 mM in 30 mM HEPES, 200 mM KCl buffer at pH 7.5. Both 2D ^1^H–^15^N experiments were collected on a 16.5-T spectrometer at 5 °C. Assignments of amide resonances for MapZ_*cyto*_ and MapZ_*cyto*_^2*TE*^ are reported in black, the mutated residues 67 and 78 are indicated in blue and red for wild-type and phosphomimetic constructs, respectively. Only minimal chemical shift differences are observed between these spectra and shifts in the cytoplasmic domain correspond solely to residues neighboring the mutated sites. In both protein constructs, residues 1 to 159 belong to the MapZ sequence, while residues 160 to 168 are reminiscent of the His-TEV site tag after TEV cleavage.

**Figure 2:**
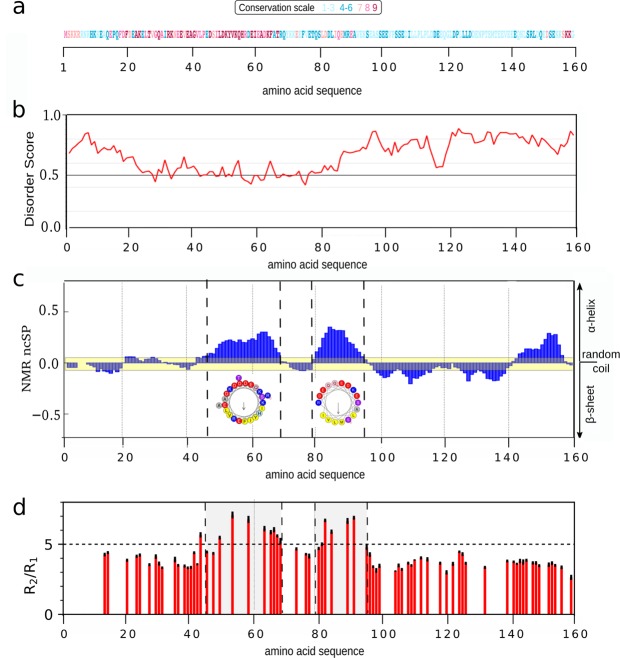
Structural organization of MapZ cytoplasmic domain (residues 1-159). (**a**) Amino acid conservation scores as calculated by the Consurf webserver (http://consurf.tau.ac.il/)^23^ are displayed on the MapZ_*cyto*_ protein sequence. Scores range from 0 (not conserved, white) to 9 (highly conserved, magenta). (**b**) Disorder scores predicted along the protein sequence by the IUPred software (http://iupred2a.elte.hu/)^19^. Highly disordered regions correspond to scores above 0.5. (**c**) Neighbor-corrected structural propensity (ncSP)^25^ scores calculated from C’, C^*α*^, and C^*β*^ NMR chemical shifts of MapZ_*cyto*_. ncSP scores reveal the propensity to form secondary structures (zero for random coil, positive for *α*-helices and negative for *β*-sheets). The yellow band represents a cutoff value of 0.1, which outlines the absence of marked structural propensity. A helix plot representation obtained from the HELIQUEST server^27^ is displayed for two regions (*π*-helix for residues 45 to 68 and *α*-helix for residues 79 to 95) with larger helical propensity. Positively-charged, negatively-charged, polar, and non-polar hydrophobic residues are colored in dark-blue, red, purple, and yellow, respectively. (**d**) Ratio of R_2_/R_1_ relaxation rate constant values (in red) and corresponding errors (in black) obtained at 5 °C displayed on the MapZ_*cyto*_ sequence. R_2_/R_1_ ratio values significantly above average are identified for regions spanning residues 45 to 68 and 79 to 95. This might suggest the presence of a transient local compactness for those two regions.

The intracellular domain of MapZ (MapZ_*cyto*_) stretches from residues 1 to 159 and can be phosphorylated at threonine 67 and 78 by the serine/threonine kinase StkP (Fig. 1a)^11,12^. To assess the structural organization of this domain (see amino acid sequence in Fig. 2a), we first overproduced and purified ^15^N-labeled MapZ_*cyto*_. The ^1^H-^15^N correlation spectrum of this MapZ_*cyto*_ construct displays typical features of an unfolded protein, *i.e*. intense narrow peaks and very low chemical shift dispersion, especially in the proton dimension (Fig. 1b). This observed unfolded state confirms the disorder score predicted from the primary sequence by the IUPred software^19^ (Fig. 2b). Indeed, the disorder score obtained from IUPred is 0.8, characteristic of an unfolded domain, except for residues 22 to 86, where the score is closer to 0.5.

The cytoplasmic domain of MapZ therefore appears to be mostly an intrinsically disordered region (IDR) with moderate tendency to be structured for the amino acid region 22 to 86. Such regions with predicted low-score disorder frequently contain “molecular recognition features” (MoRFs) that consist of an amino-acid stretch that either gains stable fold upon binding to its partner, or stays unstructured and rapidly probes the surface of the interaction partner while simultaneously switching between multiple low-affinity binding sites^20–22^. The possible participation of the N-terminal region of MapZ in protein-protein interactions is supported by the presence of a large patch of conserved amino acids extending from residues 18 to 67, as emphasized by the Consurf amino acid conservation scores (Figs. 2a and S1) obtained from multiple sequence alignment of MapZ proteins from different *Streptococcaceae*^23^.

### Structural recognition features in MapZ_*cyto*_

To further characterize the folding propensity of residues 22 to 86, backbone resonances were assigned using the standard set of 3D ^1^H,^13^C,^15^N-multidimensional NMR experiments^24^. With a more than 95% completion of backbone resonance assignment we could calculate chemical shift deviations from neighbor-corrected random coil values (extracted from the intrinsically disordered protein library^25^) for most residues and structural propensities could be deduced from these experimental data^26^. Resulting neighbor-corrected structural propensity (ncSP) values of MapZ_*cyto*_ are reported in Fig. 2c for each residue along the protein sequence.

To identify marked transient secondary structure, we looked for amino acid stretches with ncSP values above 0.25 and found three regions with helical folding propensity. Regions spanning residues 45 to 68, 79 to 95, and 142 to 156 showed ncSP values comprised between the threshold value and 0.36, 0.42, and 0.33, respectively. We screened these regions with the Heliquest server^27^ to search for potential hydrophobic patches. The first region, which contains the conserved residues 45 to 68 (Figs. 2a and S1), presents *π*-helix conformation with a face containing mainly charged residues (D46/D50/K54/E59/D63) opposite to a hydrophobic face composed of non-polar residues I48/Y52/H56/I60/F65, emphasizing its potential as an interaction interface. The second region with *α*-helical propensity (residues 79 to 95) was not predicted by the disorder score in Fig. 2b and encompasses residues that are less conserved (Fig. 2a). It contains a similar distribution of residues with a hydrophobic patch with the residues L81/I85/M88/V92 on the opposite face to a highly charged face with residues D83/E87/E90/K94. In contrast, no hydrophobic face emerges from the third region with helical propensity (residues 142 to 156), making this region a weaker candidate for molecular interaction compared to the previous two.

We also measured ^15^N-relaxation properties of MapZ_*cyto*_ backbone amide groups. The average values of the R_1_ and R_2_ relaxation rate constants were 1.7 ± 0.1 *s*^−1^ and 7 ± 2 *s*^−1^, respectively. As well, the average ^1^H-^15^N heteronuclear nOe was below 0.5 across the cytoplasmic domain of MapZ, which is typical for IDRs of similar size at 5 °C (Fig. S2)^28^. Interestingly, R_2_/R_1_ ratios remain relatively constant along the sequence except for regions spanning from residues 45 to 68 and 79 to 95, which were previously identified with modest but marked helical propensity (Fig. 2d). This relaxation heterogeneity is due to an increase in the R_2_ rate constant that could result from differences in sequential *τ*_*c*_ and the presence of a transient local compactness. Combined with the helical propensity of these two regions (Fig. 2c), this strongly supports the existence of transitory amphipathic helical secondary structures in MapZ_*cyto*_ that could promote interaction with cytoplasmic partners.

Altogether, the chemical shift and ^15^N-relaxation parameters identified two regions of local compactness with higher *α*-helical propensity for residues 45 to 68 and 79 to 95, respectively. The first region displays a patch of highly conserved residues (Figs. 2a and S1), whereas both regions display a hydrophobic face opposite to a highly charged face. The high local mobility of MapZ_*cyto*_ domain, combined with the presence of these two more compact short domains, grant it an advantage in terms of interaction with possible binding partners, notably FtsZ, which is the only known protein partner of MapZ^11^.

### Interaction of MapZ_*cyto*_ with monomeric and polymeric FtsZ

We overproduced and purified two different versions of recombinant FtsZ (see Methods section). The first version, FtsZ_*a*_, corresponded to the wild-type FtsZ from *S. pneumoniae* (strain ATCC BAA-255/R6) with no purification tag and was produced in *E. coli* C41(DE3). The FtsZ_*b*_ construct was produced in *E. coli* BL21 (DE3) and expressed from a pGEX vector encoding the glutathione S-transferase (GST)-tagged protein, whereby the N-terminal GST tag was removed by Tobacco Etch Virus (TEV) protease. The only difference between the two constructs was a glycine residue at the N-terminus coming from the tag scar in FtsZ_*b*_. ^1^H NMR spectra collected on both samples at 25 °C in the same buffer suggest that both protein constructs have the same structure (Fig. S3a).

**Figure 3:**
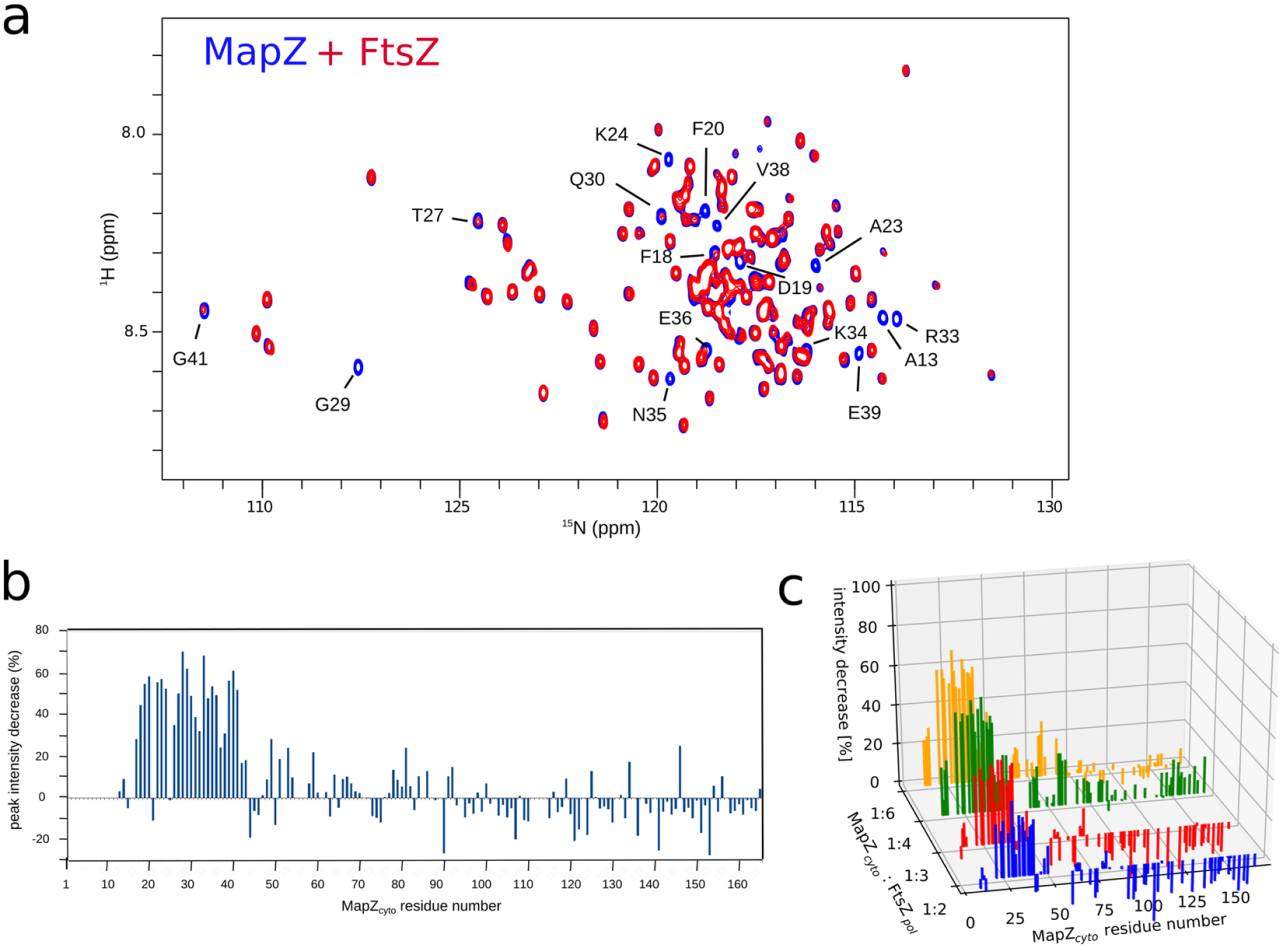
Interaction of MapZ_*cyto*_ with monomeric FtsZ. (**a**) Superimposition of the ^1^H–^15^N BEST-TROSY correlation spectra recorded on MapZ_*cyto*_ in absence (blue) and in presence (red) of 4.7 molar equivalent of FtsZ_*a*_. The samples were prepared with 88 *µ*M final concentration of ^15^N MapZ_*cyto*_ in 30 mM HEPES, 50 mM KCl buffer at pH 7.5. NMR experiments were recorded on 16.5-T Bruker Avance III spectrometer at 5 °C. (**b**) Peak intensity decrease upon addition of 4.7 molar equivalent of FtsZ_*a*_ on MapZ_*cyto*_ are reported as a function of the residue position in MapZ primary sequence. For each assigned resonance that is not significantly overlapped, the intensities I_0_ and I_1_ are measured in the reference blue spectrum containing only MapZ_*cyto*_ and in the red spectrum containing the MapZ_*cyto*_-FtsZ_*a*_ mixture, respectively, reported in (**a**). The peak intensity decrease (in %) is calculated as the $\frac{{{\rm{I}}}_{0}-{{\rm{I}}}_{1}}{{{\rm{I}}}_{0}}$ ratio. (**c**) A 3D plot reporting the peak intensity decrease as a function of the residue position in MapZ primary sequence along MapZ_*cyto*_ titration by polymeric FtsZ_*b*_ (FtsZ_*b*_). Blue, red, green and yellow histograms represent 1:1, 1:3, 1:4 and 1:6 molar ratios of MapZ_*cyto*_:FtsZ_*pol*_. Samples were prepared from 0.5 mM and 210 *µ*M stock solutions of MapZ_*cyto*_ and FtsZ_*b*_, respectively, in 30 mM HEPES, 200 mM KCl buffer at pH 7.5. The final MapZ_*cyto*_ concentration in each sample was 30 *µ*M, and 10 mM GTP and MgCl_2_ were added to polymerize FtsZ, as verified by electron microscopy.

We first investigated the interaction between MapZ_*cyto*_ and monomeric FtsZ, *i.e*. in the absence of guanosine triphosphate (GTP) and MgCl_2_. For this purpose, we recorded ^1^H,^15^N BEST-TROSY spectra on ^15^N-labeled MapZ_*cyto*_ alone or in the presence of increasing amount of unlabeled, monomeric FtsZ (up to 6-fold molar excess). Due to the tendency of FtsZ to form oligomers/bundles and polymerize at higher concentration, we used a MapZ_*cyto*_ protein concentrations below 90 *µ*M, typically in the range of 30 to 50 *µ*M. Samples used for different titration points were prepared from the same protein and buffer batches and contained the same concentration of MapZ_*cyto*_. An example of MapZ_*cyto*_ BEST-TROSY spectra collected before and after addition of 4.7 molar equivalent of FtsZ_*a*_ is shown in Fig. 3a. Strikingly, addition of monomeric FtsZ caused no chemical shift perturbation but a decrease in the intensity of some of signals. For further analysis, the decrease in signal intensity was quantified for each of the amide resonances and the values are reported along the MapZ_*cyto*_ sequence (Fig. 3b). The N-terminal residues 17 to 43 experienced an intensity decrease of up to 70% for the MapZ_*cyto*_:FtsZ_*a*_ 1:4.7 ratio, whereas other residues of the cytoplasmic domain of MapZ remained largely unaffected. Similar results were obtained with the FtsZ_*b*_ construct in the presence of which the intensity of the same signals decreased stronger at higher MapZ_*cyto*_:FtsZ_*b*_ protein ratios (Fig. S4a). These results suggest that the 17-to-43 N-terminal region of MapZ_*cyto*_ interacts with monomeric FtsZ, whereas the remainder of the cytosolic region stays completely flexible in the protein-protein complex. This conclusion differs from recently reported data by Feng *et al*.^17^, who proposed several polar residues to be involved in the MapZ-FtsZ interaction. However, the chemical shifts of these residues, which are distributed all along the amino acid sequence, are more likely induced by small variations in small ionic strength of the buffer, or residual magnesium ions coming from the FtsZ preparation. Our conclusion is consistent with our previous observations that MapZ alleles lacking the first 42 N-terminal residues were unable to promote correct FtsZ placement^11^.

We then turned to polymeric FtsZ considering that its interaction with MapZ_*cyto*_ might be dependent on the oligomeric state of FtsZ. We thus collected ^1^H,^15^N BEST-TROSY spectra after addition of GTP and MgCl_2_ to MapZ_*cyto*_:FtsZ mixtures at different molar ratios. Parallel samples were analyzed by negative-staining electron microscopy to ascertain FtsZ polymerization under the conditions used (Fig. S3b). Again, no chemical shift perturbation was observed, but resonance intensity decreased for some of the amide resonances. Data are reported in Fig. 3b for MapZcyto:FtsZ_*a*_ and in Fig. 3c for different MapZ_*cyto*_:FtsZ_*b*_ mixtures. The intensity changes along the MapZ sequence was similar compared to those observed for the monomeric form of FtsZ, suggesting that the interaction site of MapZ_*cyto*_ with monomeric or oligomeric FtsZ was essentially the same. In both cases, the overall decrease in signal intensity as a function of the MapZ_*cyto*_:FtsZ ratios suggested apparent equilibrium dissociation constants in the range of 10–100 *µ*M if one considers a 1:1 complex (which was two orders of magnitude weaker than the value reported by Feng *et al*.^17^). The apparent dissociation constant was slightly lower for the FtsZ oligomers than for the monomers (Fig. S4) and depended on the salt concentration.

**Figure 4:**
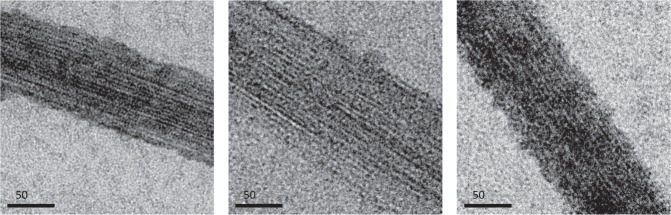
Effect of MapZ_*cyto*_ on FtsZ bundling. Negative-stain electron microscopy images of FtsZ filament bundles imaged with a calibrated nominal magnification of 23,000. FtsZ polymerization and filament bundling were performed for 15 min at room temperature in buffer at pH 7.6 containing 50 mM HEPES, 200 mM KCl, 5 mM MgCl_2_, 5 mM GTP, and 8% (wt/vol) PVA, in the absence (left panel) or in the presence of 50 *µ*M MapZ_*cyto*_ (middle panel) or 50 *µ*M BSA (right panel).

To further investigate the interaction between polymeric FtsZ and MapZ_*cyto*_, we determined the capacity of FtsZ to form filament bundles in the presence of MapZ_*cyto*_ by negative-stain electron microscopy. In a polymerization buffer containing 8% polyvinyl alcohol (PVA), FtsZ_*a*_ filaments formed bundles that were longer than 10 *µ*m and between 50 to 150 nm thick (Figs. 4 and S5). MapZ_*cyto*_ at an equimolar ratio relative to FtsZ had no effect on FtsZ bundles although the protein concentrations were 500-fold above the apparent dissociation constant of the FtsZ-MapZ interaction^11^. When MapZ_*cyto*_ and FtsZ were mixed at a 10:1 ratio, there was no major effect on the FtsZ bundles at high magnification (Fig. 4), but FtsZ bundles and isolated FtsZ filaments seemed less abundant (Fig. S5), consistent with a previous study by Feng *et al*. who reported that the addition of MapZ destabilizes the bundling of FtsZ polymers^17^. However, we observed a similar destabilization effect when bovine serum albumine (BSA) was used instead of MapZ (Figs. 4 and S5). On the other hand, in the absence of PVA, an equimolar or 10:1 MapZ_*cyto*_:FtsZ ratio did not promote FtsZ bundling. Altogether, these observations suggest that the formation of stable FtsZ filaments and their bundling are affected by high protein concentrations, but are not specifically affected by the presence of MapZ_*cyto*_. However, we cannot exclude that the modulation of FtsZ filament formation and/or bundling by MapZ might be enhanced by the presence of a yet unknown protein partner.

Our NMR and EM data show that a relatively low affinity complex forms between MapZ_*cyto*_ and monomeric as well as polymeric FtsZ. Only the 17-to-43 residue stretch at the N-terminus of MapZ interacts with FtsZ. This highly conserved region contains MoRFs that remain largely unstructured and rapidly probe the surface of its FtsZ binding partner. This functional role of MapZ could be crucial, as the complex needs to be formed transiently at different stages of the division. This hypothesis is also consistent with the findings that MapZ serves both for initial positioning of FtsZ at mid-cell, suggesting an interaction with FtsZ monomers, and for the regulation of the Z-ring constriction, suggesting an interaction with FtsZ bundles^11^.

### Interaction of MapZ_*cyto*_ with liposomes that mimic the cell membrane of *S. pneumoniae*

**Figure 5:**
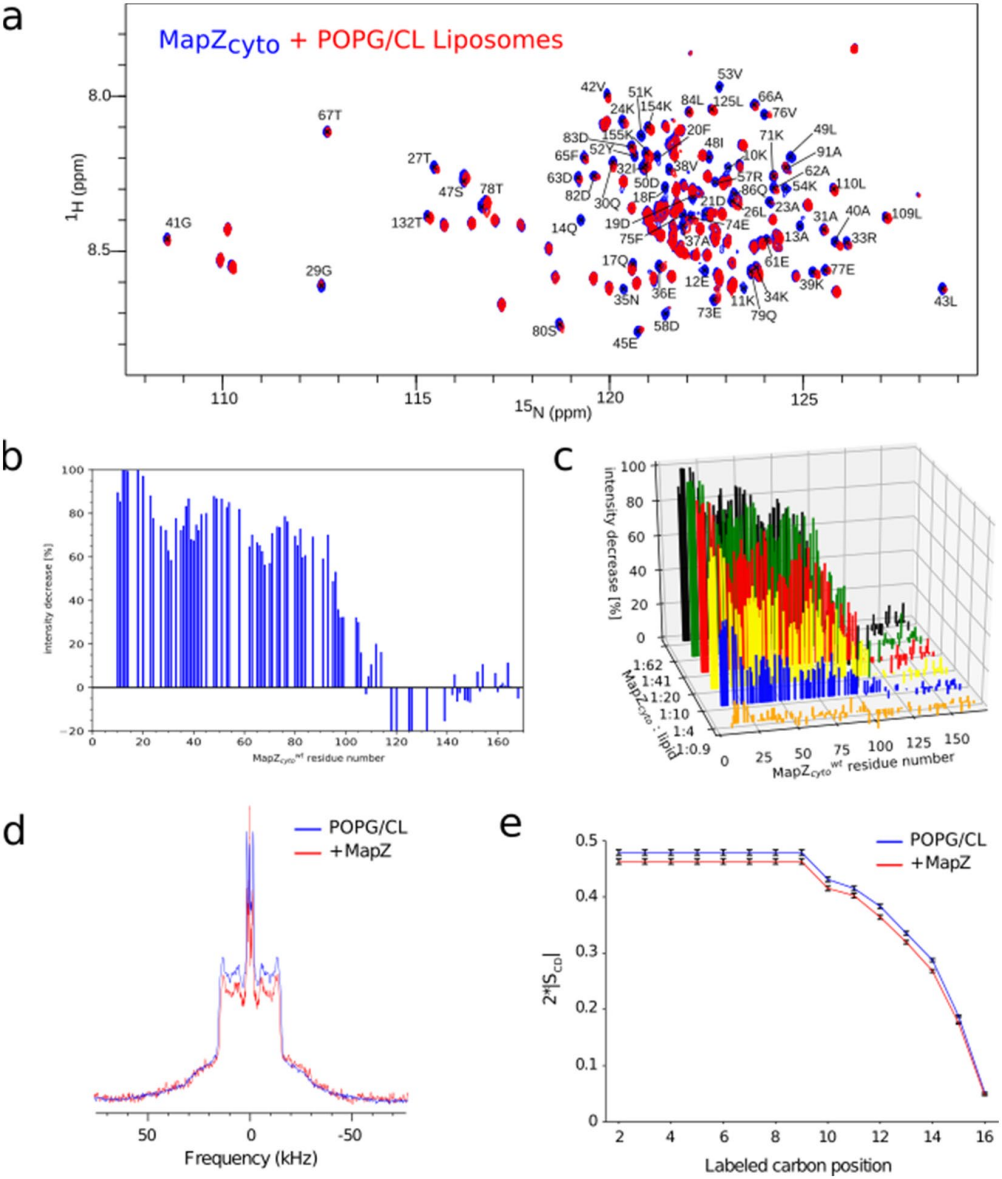
Interaction between MapZ_*cyto*_ and liposomes. (**a**) ^1^H–^15^N BEST-TROSY spectra of ^15^N-labeled MapZ_*cyto*_ recorded in the absence (blue) and in the presence (red) of 50-nm liposomes composed of an equimolar mixture of POPG and CL. (**b**) Decrease in (%) of the MapZ_*cyto*_ resonance intensity for each amide resonance. (**c**) Graph showing the intensity decrease in (%) for each MapZ_*cyto*_ amide resonance in the ^1^H–^15^N correlation spectra at different protein:lipid molar ratios. Data in (**a**–**c**) were collected on a 100 *µ*M sample of ^15^N MapZ_*cyto*_ in 50 mM Tris, 150 mM NaCl buffer at pH 7.5. The stock solution of SUVs was prepared in the same buffer with a 54 mM lipid concentration. Data in (**a**,**b**) corresponds to a 1:62 MapZ_*cyto*_:lipid molar ratio. NMR experiments were recorded on a 22.4-T spectrometer at 5 °C. Panels (a) through (c) point to an interaction between SUVs and the N-terminal domain of MapZ_cyto_. (**d**) Wide-line (static) ^2^H-ssNMR spectra of PG-d62:CL deuterated multilamellar vesicles (MLVs with a 1:1 molar ratio of both lipids) in the absence (blue) or presence (red) of MapZ_*cyto*_ at a lipid:protein molar ratio of 50:1. (**e**) Value of the carbon–deuterium order parameters |S_CD_|, obtained from back-calculated ^2^H-SSNMR spectra of deuterium-labeled phosphoglycerol lipid PG-d62 in the liposomes, in the absence (blue) and in the presence (red) of MapZ_*cyto*_ are plotted as a function of the carbon position along the phosphoglycerol lipid chain. Panels (d,e) evidence a slight reduction of the liposome cohesion.

Since MapZ_*cyto*_ domain is an intrinsically disordered domain that is anchored to the cell membrane in the context of the full-length protein, we investigated its capacity to interact with a membrane-like environment. For this purpose, we first considered small unilamelar vesicles (SUVs) with a lipid composition that is similar to the composition of the cytoplasmic membrane of *S. pneumoniae*. These monodisperse 50-nm wide liposomes consisted of a mixture of two lipids with negatively charged polar-heads, 1-palmitoyl-2-oleoyl-sn-glycero-3-phospho-(1′-rac-glycerol) (POPG) and cardiolipin (CL), mixed in a 1:1 molar ratio^29^. Their interaction with MapZ_*cyto*_ was studied using NMR spectroscopy and ^1^H–^15^N BEST-TROSY correlation spectra of ^15^N- MapZ_*cyto*_ were recorded after addition of different molar ratio of SUVs (one SUV contains approximately 7000 lipid molecules). Consistent with previously described experiments on the interaction between MapZ_*cyto*_ and FtsZ, neither new signals nor chemical shift perturbations of existing resonances were observed for a 1:62 MapZ_*cyto*_:lipid molar ratio (Fig. 5a). Instead, some of the resonances showed decreased intensity after addition of liposomes to MapZ_*cyto*_ (Fig. 5a,b). This decrease even exacerbated with the addition of higher concentration of liposome (Fig. 5c) and the affected residues localized at the N-terminus of the cytoplasmic domain of MapZ, as shown in Fig. 5b,c, demonstrating a clear interaction between the lipids and the protein.

Complex formation between MapZ_*cyto*_ and SUVs was further confirmed by DOSY NMR experiments (see Supplementary Information). Indeed, this type of experiment provides an estimate for the translational diffusion coefficient of the various components of biological samples, as this coefficient is sensitive to differences in molecular weight. We focused on the resolved methyl protons of the protein and used these to determine the diffusion coefficient along the titration of MapZ_*cyto*_ with SUVs. As shown in Fig. S6, we observed a decrease in the diffusion coefficient D with increasing concentrations of SUVs in the sample (the value of D diminished by a factor up to 3 for a MapZ:lipid molar ratio of 1:62). This observation is consistent with the formation of a protein-SUV complex in rapid exchange, i.e. a rapid interconversion between the free and complexed protein forms.

When intensity perturbations were analyzed in more detail at a high lipid to MapZ_*cyto*_ ratio (Fig. 5b), residues 11 to 110 show values of the intensity decrease that are above 20%, with lower values towards the end of this region. This result suggests that, in the presence of SUVs, the C-terminal part of MapZ_*cyto*_ (residues 110 to 159) remains flexible upon interaction of the N-terminal part (residues 11 to 110) with the liposomes. Interestingly, this N-terminal region contains the two previously identified portions with helical propensities (residues 45 to 68 and 79 to 95 (Fig. 2c)). Interaction between MapZ_*cyto*_ and liposomes could thus be mediated by the formation of these amphipathic helices, the first of them being highly conserved in MapZ proteins of all *Streptococcaceae*. The N-terminal region of MapZ_*cyto*_ is also characterized by the presence of positively charged amino acids (residues 3 to 11) that may promote the interaction with the negatively charged phosphate groups of the POPG and cardiolipin heads while remaining largely unfolded.

To gain further information on protein-lipid interaction and the associated modifications of membrane structure and dynamics, we performed static ^2^H solid-state NMR (ssNMR) experiments. The quadrupolar splitting of the ^2^H nucleus in ^2^H 1D ssNMR spectra directly reports on the phase behaviour of the deuterated lipid and on the lipid acyl chain deuterium-carbon order parameter for all deuterated methylene groups of the acyl chain, reflecting the membrane dynamics^30,31^. The so-called De-Pake-ing^32^ decodes the quadrupolar ^2^H doublet into local order parameter |2*S_*CD*_| for each deuterium-carbon bond of the methylene groups of the lipid acyl chain. Here we used deuterated 1,2-dipalmitoyl-d62-sn-glycero-3-[phospho-rac-(1-glycerol)] (PG-d62) doped with cardiolipin (CL) (in a 1:1 molar ratio) to reflect the association of MapZ_*cyto*_ on negatively charged lipids. An interaction between membrane-associating proteins with the relevant membrane lipids leads to significant modifications in the dynamics of membrane lipids as reported by the deuterium quadrupolar splitting and the methylene order parameters. Figure 5d shows a slight modification of the quadrupolar splitting pattern of the deuterium signal from all methylene groups of the lipidic acyl chains. This is further evidenced in Fig. 5e after deconvolution of the deuterium signal and calculation of the carbon-deuterium order parameters for each of the methylene groups of the palmitoyl chain. MapZ_*cyto*_ influences the membrane structural and dynamical behavior by more significantly lowering the order parameter |2*S_CD_| for the methylene groups that are closer to the polar heads of the lipids (carbon 1 through 9). Discernible effects can nevertheless be perceived up to the inner carbon atoms (carbon 14). The decrease in order parameter reflects the increase in the acyl chain mobility in the liposome, suggesting a fluidifying effect of MapZ_*cyto*_ on PG/CL-containing membranes.

Altogether, monitoring of MapZ_*cyto*_ with liquid-state NMR and deuterated liposomes with ^2^H 1D ssNMR shows that the N-terminal portion of MapZ_*cyto*_ (up to residue 110) interacts with membrane lipids. On one hand, the unfolded and positively charged region of MapZcyto (residues 3 to 11) interacts with the negatively charged lipid heads. On the other hand, the two amphipathic helices (residues 45 to 68 and 79 to 95), which present hydrophobic spots surrounded by positively charged and hydrophilic residues, induce packing defects of the lipids by increasing the mobility of their acyl chains^33^. The interaction of the cytoplasmic domain of MapZ with the membrane may be regulated on one side by the membrane composition and curvature and on the other side by the competitive interaction with FtsZ, as the regions of MapZ_*cyto*_ interacting with liposomes and FtsZ overlap. The versatility of this interaction could be essential to the regulatory role of MapZ in the Z-ring positioning.

### Phosphomimetic MapZ_*cyto*_

MapZ is phosphorylated by StkP at T67 and T78. To determine whether phosphorylation affects the structural and dynamical behavior of MapZ_*cyto*_, we produced and purified a ^13^C- and/or ^15^N-labeled phosphomimetic mutant of MapZ_*cyto*_ (MapZ_*cyto*_^2*TE*^) in which T67 and T78 are replaced with glutamic acid. The ^1^H-^15^N correlation spectrum of MapZ_*cyto*_^2*TE*^ displayed typical features of an unfolded protein and superimposed well with the MapZ_*cyto*_ spectrum (Fig. 1b) except at the proximity of mutated residues. Backbone resonance assignments were transferred from MapZ_*cyto*_ to MapZ_*cyto*_^2*TE*^ and completed with a limited set of 3D NMR experiments to the same level of completion as the wild type protein. Analyses of the assigned resonances of MapZ_*cyto*_^2*TE*^, similar to those performed on the wild-type construct, provided a similar secondary structure propensity profile (Fig. S7). This strongly supports the notion that phosphorylation has minimal effect on the structural and dynamic behavior of MapZ_*cyto*_ in the absence of interaction partners and that MapZ_*cyto*_^2*TE*^ remains an intrinsically disordered domain.

To investigate the effect of MapZ phosphorylation on the molecular interaction with FtsZ, we titrated the phophomimetic mutant MapZ_*cyto*_^2*TE*^ with monomeric and polymeric FtsZ. A slightly different protocol was adopted for the sample preparation, *i.e*. diluted MapZ and FtsZ protein solutions were mixed at the desired molar ratio before the protein mixture was concentrated to a final MapZ concentration of 100 *µ*M. The same protocol was repeated with wild-type MapZ_*cyto*_ for direct comparison. Titration results are reported in Fig. S8a–c. The regions of MapZ_*cyto*_^2*TE*^ that were the most affected by the monomeric or polymeric forms of FtsZ include a large region spanning residues 21 to 45 and a narrow one centered on residue 81 (Fig. S8a,b). Similar observations were made when the wild-type construct of MapZ_*cyto*_ was mixed with FtsZ (Fig. S8c). Altogether, these results are consistent with the previous observation that phosphorylation does not affect interaction with FtsZ^11^.

We next investigated the interaction of MapZ_*cyto*_^2*TE*^ with liposomes. The profiles of the resonance intensity decrease along the sequence of MapZ_*cyto*_ showed that lipids similarly affected the N-terminal region of the wild-type and 2TE constructs, with a minimal effect after residue 100 (Fig. S8d,e). Only regions in the vicinity of the mutations displayed a larger peak intensity decrease value than in the corresponding regions of the wild type construct, suggesting that phosphorylation may locally induce a stronger interaction with the SUVs. These *in vitro* data obtained with the phosphomimetic mutant of MapZ_*cyto*_ are in agreement with previous *in vivo* studies, which proposed an indirect effect of phosphorylation on cell division since MapZ phosphomimetic and phosphoablative variants retained septal localization and did not alter the Z-ring positioning^11,12^.

## Conclusion

Usually, proteins fold into a unique and stable three-dimensional structure before becoming biologically active. However, studies over the last decade have provided convincing evidence that intrinsically disordered proteins (IDPs) do not adopt a single structure despite being fully functional. High local flexibility of IDPs allows for binding promiscuity and the possibility to interact with many binding partners. The presence of several Molecular Recognition Features (MoRFs) within a sequence of a single IDP allows cells to create molecular protein hubs that serve within cell signaling and regulatory processes^34,35^. We have shown in this study that the cytoplasmic domain of MapZ belongs to this family of intrinsically disordered domains.

In the absence of its natural partners, MapZ_*cyto*_ is mainly unstructured except for two specific regions (45–68 and 79–95) that show low but marked propensities to form amphipathic helical structures. Our data suggests that the interaction with FtsZ, which is mainly established by residues 17 to 43 in MapZ, does not affect the structural features of the rest of the protein. This interaction is insensitive to the polymerization status of the FtsZ protein. Consistent with these observations, but in contrast with a recent publication^17^, we did not detect any specific effect of MapZ on FtsZ polymerization or bundling. If MapZ impacts the formation of FtsZ bundles, it might only rely on a variation of local MapZ concentration and crowding, or require the presence of a third yet-to-be identified protein partner.

**Figure 6:**
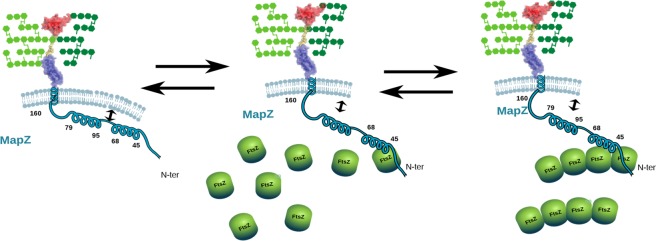
Different states for MapZ and its interaction partners: a complex equilibrium *in vivo*. The cytoplasmic membrane, the FtsZ monomers, the peptidoglycan, are shown in light blue, as green balls, and as green cross-linked hexagons, respectively. The structural features of MapZ are reported with the ribbon structures of the two extracytoplasmic domains determined by NMR connected with the flexible serine-rich linker in yellow and with the transmembrane and cytoplasmic transient amphipathic helices schematized in blue. Interaction sites and complex formation are visualized through spatial proximity.

In addition, we revealed that MapZ_*cyto*_ can interact with the surface of liposomes formed with POPG/PG and cardiolipin mimicking the *S. pneumoniae* cytoplasmic membrane. Furthermore, the association of MapZ to PG/CL-containing membranes increases the membrane fluidity. The region of MapZ that interacts with liposomes completely overlaps the region that interacts with FtsZ and extends up to residue 110. As a result, the MapZ-lipid interaction site contains the two identified amphipathic helices transiently forming in the free form, with the first one located in the highly conserved region ranging from residues 18 to 67. Recruitment of FtsZ at mid-cell by the N-terminal region of MapZ could thus be modulated by the availability of this flexible extremity, which is in competition with the membrane for interaction. The outcome of this interaction could be modulated by the curvature or/and the composition of the membrane at mid-cell (Fig. 6). Conversely, MapZ binding to FtsZ may abolish the interaction of MapZ with lipids, thus modifying the fluidity of the membrane at the division site. This change could constitute a step required for cell constriction although the interaction of MapZ with lipids and FtsZ may not be mutually exclusive. Indeed, the region interacting with liposomes only partially overlaps with that of FtsZ, with the region including the two amphipathic helices (residues 45–110) being only involved in lipid binding. In this scenario, the tripartite complex could be an initial step toward the assembly of the divisome. In further support of this idea, it was recently shown that the localization of liquid and gel lipid phases correlates with the localization of FtsZ and the lipid II precursor of PG^36^.

Finally, we showed that MapZ phosphorylation affects neither its structural organization, nor its interaction with lipids and FtsZ. However, MapZ phosphorylation affects the cell division process^11^. These observations indicate that other partners of MapZ, whose function would be affected by or dependent on MapZ phosphorylation, remain to be identified. Thus, we are still merely at the tip of the iceberg in our understanding of the intricacies of the MapZ system. Complicated as it may be, understanding the molecular dialog occurring at mid-cell between MapZ, FtsZ, lipids and other partners of the divisome is essential to depict the mechanism of cell division in the pneumococcus.

## Methods

### Production and purification of MapZ_*cyto*_ constructs

The pEtPhos vectors encoding wild-type or phophomimetic T67E/T78E double-mutant of the cytoplasmic domain of MapZ (from residue 1 to 159) followed by a three-amino-acid (LQG) linker, a Tobacco Etch Virus (TEV) protease cleavage site (ENLYFQRGG), and a hexa-histidine tag (adapted from Supplementary Tables 1 and 2 in Fleurie *et al*.^11^) were used to produce *S. pneumoniae* (strain ATCC BAA-255/R6) MapZ_*cyto*_ or MapZ_*cyto*_^2*TE*^ samples, respectively. ^15^N- or ^13^C,^15^N-isotopically labeled MapZ_*cyto*_ and MapZ_*cyto*_^2*TE*^ were expressed in *E. coli* BL21 (DE3) strain, grown at 37 °C in M9 medium^16^ supplemented with ^13^C-labeled glucose or/and ^15^NH_4_Cl, as well as ampicillin (100 *µ*g/mL). Protein production was induced with 1 mM isopropyl-*β*-D-thiogalactopyranoside (IPTG) when the culture reached OD_600_ = 0.6. After 3 hours of expression cells were collected and frozen at −20 °C. For both proteins, the cell pellet was resuspended in 25 mL of lysis buffer (50 mM Tris, 500 mM NaCl, 10 mM imidazole, 30 *µ*g/mL DNase, 30 *µ*g/mL RNase, 1 tablet of cOmplete protease inhibitor cocktail, pH 7.5). After sonication (10 min of 2s-on-8s-off cycles with an amplification of 60% on a Branson Ultrasonics apparatus) and centrifugation (46,000 × g, 30 min, 4 °C), the supernatant was loaded on a Ni-NTA column (Qiagen). Fractions containing the overexpressed protein were pooled and dialyzed overnight at 4 °C against a 50 mM Tris, 100 mM NaCl, pH 7.5 buffer. The hexa-histidine tag was cleaved off by incubating with of a solubility-enhanced hexahistidine-tagged L56V/S135G TEV protease^37^ in a 20:1 (w:w) ratio for 3 hours at room temperature. The uncleaved protein and TEV protease were separated from the cleaved protein through immobilized metal affinity chromatography (IMAC) on a Ni-NTA column (Qiagen). The flowthrough containing the cleaved enzyme was concentrated and loaded onto a HiLoad 16/600 Superdex 75 (GE Healthcare) equilibrated in 30 mM HEPES, 200 mM KCl buffer at pH 7.5 for size exclusion chromatography purification. Fractions containing the pure protein were pooled and concentrated. In order to stabilize the proteins, the samples were incubated for 15 min at 80 °C before storage. Protein purity was controlled by SDS-PAGE. For long term storage, samples were rapidly frozen in liquid nitrogen and stored in a −80 °C freezer.

### Production and purification of *S. pneumoniae* FtsZ

The pMKV18 plasmid, which encodes for the *S. pneumoniae* FtsZ protein without any purification tag was a gift of Dr German Rivas. The corresponding FtsZ_*a*_ was expressed and purified as previously described^38^.

The pGEX vector encoding glutathione S-transferase (GST) followed by a short linker (LVPRGS), the TEV protease cleavage site and the wild-type FtsZ from *S. pneumoniae* was kindly provided by Dr Cecile Morlot. The GST-FtsZ protein was expressed and purified as previously described^39^. In brief, the fusion protein was expressed in *E. coli* BL21 (DE3) strain, grown at 37 °C in LB medium supplemented with 100 *µ*g/mL ampicillin. When the culture reached OD_600_ = 0.7, the expression was induced with 0.5 mM IPTG and the culture was further incubated overnight at 25 °C. Harvested cells were resuspended in 30 mL of lysis buffer (50 mM Tris, 200 mM NaCl, 1 tablet of the cOmplete protease inhibitor cocktail, pH 8.0). After sonication (10 min of 2s-on-8s-off cycles with an amplification of 60% on a Branson Ultrasonics apparatus) and centrifugation (46,000 × g, 30 min, 4 °C), the supernatant was loaded on a 5 mL Glutathione Sepharose 4B (GST) affinity column (GE Healthcare, Little Chalfont, United Kingdom). After extensive washing, the GST resin with bound GST-FtsZ was resuspended in 10 mL of washing buffer (50 mM Tris, 200 mM NaCl, pH 8.0) and 1 mg of the hexahistidine-tagged L56V/S135G TEV protease^37^ was added. The mixture was stirred for 3 hours at room temperature and the cleaved FtsZ protein was separated from the hexahistidine-tagged TEV on a Ni-NTA column (Qiagen). The fractions in 50 mM Tris, 200 mM NaCl, pH 8.0 containing 0 to 20 mM imidazole were collected, analyzed by gel electrophoresis and concentrated. The final size exclusion chromatography purification step was performed on a HiLoad 16/600 Superdex 200 (GE Healthcare) column equilibrated in 30 mM HEPES, 200 mM KCl buffer at pH 7.5. Fractions containing pure FtsZ_*b*_ protein were pooled and concentrated and protein purity was controlled by SDS-PAGE.

FtsZ_*a*_ and FtsZ_*b*_ only differ in their sequence by the additional glycine scar of the TEV cleavage site at the N-terminus of FtsZ_*b*_. To control the correct folding of FtsZ, final protein preparations were systematically checked using ^1^H 1D-sculpting NMR experiments^40^ and signal dispersion of the amide and methyl resonances outside of the [7.5, 8.5] and [0.5, −0.5] ppm window, respectively, were ascertained (Fig. S3a). In addition, negative-staining electron microscopy (Fig. S3b) was used to monitor the monomeric *versus* polymeric state of the FtsZ protein (see electron microscopy subsection below). Polymerization of FtsZ and formation of FtsZ bundles were triggered by the addition of 5 mM guanosine triphosphate (GTP) and MgCl_2_.

### Preparation of liposomes

Unlabeled liposomes for interaction studies with ^13^C,^15^N-labeled MapZ_*cyto*_ in liquid-state NMR were prepared as follows. Solutions of the sodium salts of [(2 R)-1-[2,3-dihydroxypropoxy(hydroxy)phosphoryl]oxy-3-hexadecanoyloxypropan-2-yl] (Z)octadec-9-enoate (1-palmitoyl-2-oleoyl-sn-glycero-3-phospho-(1′-rac-glycerol), POPG, Avanti Polar Lipids Inc.) and [(2 R)-3[[3-[[(2 R)-2,3-di[(9Z)-9-octadecenoyloxy]propoxy]-hydroxyphosphoryl]oxy-2-hydroxypropoxy]-hydroxyphosphoryl]oxy-2-[(9Z)-9-octadecenoyloxy]propyl] (9Z)-9-octadecenoate (1′,3′-bis[1,2-dioleoyl-sn-glycero-3-phospho]-glycerol, 18:1 cardiolipin, CL, Avanti Polar Lipids Inc.) in chloroform were mixed together in a 1:1 molar ratio in round-bottomed glass flask. Chloroform was evaporated under a constant nitrogen gas flow until a thin lipid film formed. This dried lipid film was dissolved in an appropriate quantity of 50 mM Tris, 100 mM NaCl buffer, pH 7.5 for the targeted lipid concentration. The resulting heterogeneous mixture of multilamellar vesicles was homogenized with the Avanti Mini Extruder (Avanti Polar Lipids Inc., Alabaster, USA). The mixture was pressed through a polycarbonate membrane with defined pore diameter, after 10 cycles the membrane was exchanged for a new one with smaller pore size. Membranes with pore sizes of 800, 400, 200, 100 and 50 nm were sequentially used to obtain small unilamellar vesicles (SUVs) with diameter of 50 nm. The homogeneity of the final solution and 50-nm size of POPG:CL SUVs were subsequently controlled by diffusion light scattering (DLS) on a Zetasizer Nano S instrument from Malvern Panalytical.

Deuterated liposomes for interaction studies with ^15^N-labeled MapZ_*cyto*_ in solid-state NMR were prepared from powders of deuterated 1,2-dipalmitoyl-d62-sn-glycero-3-[phospho-rac-(1-glycerol)] (DPPG-d62, Avanti Polar Lipids, Inc.) and unlabeled CL. The two lipids were mixed in a 1:1 molar ratio and dissolved in chloroform. The solvent was evaporated and redissolved as described for unlabelled liposomes and homogenized by three cycles of vortexing, flash freezing in liquid nitrogen for 1 min, and thawing for 10 min at 40 °C in a water bath. This protocol generated a milky suspension of micrometer-sized PG-d62:CL multilamellar vesicles (MLVs).

### NMR resonance assignments of MapZ_cyto_ protein constructs

The 2D- and 3D-NMR experiments were collected on 200 *µ*M ^13^C,^15^N-labeled MapZ_*cyto*_ and MapZ_*cyto*_^2*TE*^ NMR samples in 30 mM HEPES, 200 mM KCl buffer at pH 7.5 containing 10% D_2_O. Backbone resonance assignments were carried out using a combination of 2D ^1^H-^15^N-BEST-TROSY and 3D HN(CO)CACB, iHNCACB, HNCO, HN(CA)CO, H(NCACO)NH experiments. In order to limit the number of overlaps, BEST-TROSY version of the above listed experiments was used^24,41^. For the assignment of side-chains aliphatic carbons, 2D ^1^H-^13^C-HSQC and 3D (H)C(CO)NH, (H)CCH-TOCSY, and H(C)CHTOCSY experiments were collected. All spectra were recorded at 5 °C using Bruker AVANCE spectrometers operating at 700 and 850 MHz proton frequency equipped with TCI cryoprobes.

NMR spectra were processed using the TopSpin software by Bruker in its 3.2 version and were analyzed using the CcpNmr Analysis software^42^. The ^1^H chemical shifts were referenced to the internal standard 4,4-dimethyl-4-silapentane-1sulfonic acid (DSS) methyl resonance. ^13^C and ^15^N chemical shifts were referenced indirectly using the IUPAC-IUB protocol^43^. Amide, carbonyl, alpha-carbon and beta-carbon resonances could be assigned in MapZ_*cyto*_ with a 95.6%, 96.4%, 97.0%, and 96.9% completion, respectively. For this construct, 73.3% and 78.3% of the total aliphatic carbon and proton atoms, respectively, could be assigned, while aromatic side-chains remained too severely overlapped for specific assignment. Backbone resonance assignments were transferred to the phosphomimetic mutant of MapZ, MapZ_*cyto*_^2*TE*^, for most resonances. A 3D ^15^N-TOCSY-HSQC and a 2D ^13^C-HSQC were used to identify resonances from the E67 and E78 mutated residues and their neighbors. Assignment completion was identical to that of the wild-type protein.

### Liquid-state NMR titration experiments

The tendency of FtsZ to polymerize at higher concentrations, even in the absence of GTP, posed the limit on the maximum concentration of the stock solution of FtsZ to be used in titration experiments with monomeric FtsZ (concentration had to be lower than 250 *µ*M). Thus, NMR titration experiments could not be performed in the classical manner by adding a small amount of highly concentrated FtsZ solution into the MapZ NMR sample. Instead, for each MapZ_*cyto*_:FtsZ molar ratio, an individual NMR sample was prepared by mixing stock solutions of ^15^N-labeled MapZ_*cyto*_ and unlabeled FtsZ such that concentration of ^15^N-labeled MapZ_*cyto*_ was identical over all NMR samples of the titration and in the 30-to-50 *µ*M concentration range. ^1^H-^15^N BEST-TROSY spectra were then recorded to map changes in MapZ_*cyto*_ signals as induced by the presence of FtsZ. Subsequently, the effect of the FtsZ polymerization state on the interaction with the MapZ_*cyto*_ was studied. The polymerization of FtsZ was triggered by the addition of 10 mM GTP and 10 mM MgCl_2_ (using 100 mM GTP and 1 M MgCl_2_ stock solutions) to the NMR samples prepared in the previous step and containing both proteins, FtsZ and MapZ_*cyto*_. Samples were then incubated for 5 minutes at room temperature before ^1^H-^15^N BEST-TROSY data collection. The same protocol was followed to study the interaction of MapZ_*cyto*_^2*TE*^ with monomeric or polymeric FtsZ.

For NMR titration experiments with 50 nm SUVs, the concentrated aqueous solution of the POPG-CL SUVs (54 mM lipid solution in 50 mM Tris, 150 mM NaCl buffer at pH 7.5) was gradually added to the 100 *µ*M ^15^N MapZ_*cyto*_ NMR sample until complete disappearance of several MapZ_*cyto*_ signals in the ^1^H-^15^N BEST-TROSY spectra was observed. The same protocol was followed to study the interaction of MapZ_*cyto*_^2*TE*^ with 50-nm POPG:CL SUVs.

All ^1^H-^15^N BEST-TROSY experiments were recorded at 5 °C using Bruker AVANCE spectrometers equipped with a TCI cryoprobe and operating at 600, 700 or 950 MHz proton frequency. Spectra were processed using Bruker Topspin 3.2 and analyzed within CcpNmr Analysis software. Peaks were peaked automatically to determine the optimized peak position and peak intensities were calculated after gaussian lineshape fitting of each 2D-resonance. Resonance intensity decrease values (in %) were calculated as follows. For each resonance, peak intensity I_0_ was measured in the reference BEST-TROSY spectrum of MapZ_*cyto*_ in the absence of interactant and peak intensity I_1_ of the same resonance was measured in the BEST-TROSY spectrum of MapZ_*cyto*_ in the presence of interactant. These values were corrected with the dilution factor of MapZ_*cyto*_ in the corresponding experiments and the intensity decrease value in % was calculated as 100 × (I_0_ − I_1_)/I_0_. 3D plots of the titration experiment results were generated using in-house written python scripts and intensity files exported from CcpNmr.

### Solid-state NMR studies of MapZ_*cyto*_ interaction with deuterated liposomes

Static solid-state ^2^H NMR experiments were performed on a Bruker Avance II 500 MHz WB (11.75 T) spectrometer. Samples were equilibrated for 30 min at 298 K before data acquisition. ^2^H NMR experiments on PG-d62 samples were performed at 76 MHz with a phase-cycled quadrupolar echo pulse sequence (90_*x*_-*τ*-90_*y*_-*τ*-acq). Acquisition parameters for ^2^H spectra were as follows, 500-kHz spectral width, 2.90-ms *π*/2 pulse width, 40-ms inter-pulse *τ* delay, and 2-s recycling delay. Spectra were collected with 2048 scans and processed using a 200-Hz Lorentzian line before Fourier transform in the Bruker Topspin 3.2 software. Spectra deconvolution and simulation were applied to determine accurately the experimental quadrupolar splitting. Orientational order parameters (S_*CD*_) were derived from these values^44^,^45^. ^2^H solid-state NMR spectra were recorded on the micrometer-sized PG-d62:CL multilamellar vesicles (MLVs) in the presence or absence of MapZ_*cyto*_ at a lipid:protein molar ratio of 50:1 for a total lipid concentration of 35 mM. For the MapZ_*cyto*_ containing sample, the protein was added to the preformed liposomes and incubated during 30 min at room temperature.

### FtsZ polymerization and bundling assays followed by electron microscopy

To check the polymeric state of the FtsZ samples, the purified FtsZ protein was diluted to 5 *µ*M in 30 mM HEPES buffer at pH 7.6 containing 200 mM KCl, 5 mM MgCl_2_ and incubated for 15 minutes at room temperature in the presence or in the absence of 5 mM GTP. For bundling assays of FtsZ, purified recombinant FtsZ_*a*_ was diluted to 5 *µ*M in 50 mM HEPES buffer at pH 7.6, containing 5 mM MgCl_2_, 200 mM KCl, 5 mM GTP, and 8% (wt/vol) PVA. This sample was incubated for 15 min at room temperature either in the absence of any protein, in the presence of 5 or 50 *µ*M purified recombinant MapZ_*cyto*_, or in the presence of 50 *µ*M commercial BSA (Sigma Aldrich) as a control. The BSA powder was resuspended in the MapZ_*cyto*_ buffer (*i.e*. 30 mM HEPES, 200 mM KCl, pH 7.5). In the experiment performed without MapZ_*cyto*_ or BSA, proteins were replaced by an equivalent volume of MapZ_*cyto*_ purification buffer. For electron microscopy analysis, negative stain Mica-carbon Flotation Technique (MFT) was used to prepare samples^46^. In brief samples were absorbed on the clean side of a carbon film on mica, stained with 2% (wt/vol) uranyl acetate. Samples were then transferred to a 400-mesh copper grid, which was subsequently air-dried. Images were taken under low dose conditions (<10 e^−^/Å ^2^) with defocus values between 1.2 and 2.5 *µ*m on a Technai 12 FEI LaB6 electron microscope at 120 kV accelerating voltage. Image acquisition was performed with calibrated nominal magnifications ranging from 440 to 23,000, using a CCD Gatan ORIUS SC1000 camera (Gatan, Inc., Pleasanton, CA).

## Supplementary information

Supplementary information.
