# Supplementary material for: Structural features of the interaction of MapZ with FtsZ and membranes in Streptococcus pneumoniae
Source: Sci Rep. 2020 Mar 4;10:4051. doi: 10.1038/s41598-020-61036-9 (PMC7055233; doi:10.1038/s41598-020-61036-9)
Supplement: Supplementary file 1 — Supplementary information. [file 41598_2020_61036_MOESM1_ESM.pdf]

SUPPLEMENTARY INFORMATION

Structural features of the interaction of MapZ with FtsZ and membranes in *Streptococcus pneumoniae*

Tomas Hosek[1], Catherine M. Bougault[1], Jean-Pierre Lavergne[2], Denis Martinez[3], Isabel Ayala[1], Daphna Fenel[1], Marine Restelli[2], Cécile Morlot[1], Birgit Habenstein[3], Christophe Grangeasse[2], and Jean-Pierre Simorre[1,*]

[1] Univ. Grenoble Alpes, CNRS, CEA, Institut de Biologie Structurale, F-38000 Grenoble, France

[2] Molecular Microbiology and Structural Biochemistry, CNRS UMR 5086, Université de Lyon, Lyon, France

[3] Institute of Chemistry and Biology of Membranes and Nano-objects, CBMN-CNRS Université de Bordeaux, Pessac, France

*jean-pierre.simorre@ibs.fr

Table of contents

Supplementary Material and Methods **3**

Supplementary Figures **4**

Supp. Figure S1: Multiple sequence alignment of the cytoplasmic domain of MapZ used to calculate conservation scores with Consurf 4

Supp. Figure S2: Relaxation data for backbone amide ^{15}N-resonances of $MapZ_{cyto}$ 5

Supp. Figure S3: Control of FtsZ folding by NMR and polymerization state by electron microscopy 6

Supp. Figure S4: Interaction of $MapZ_{cyto}$ with monomeric and polymeric $FtsZ_b$ 7

Supp. Figure S5: Effect of $MapZ_{cyto}$ on FtsZ bundling 8

Supp. Figure S6: Evolution of the translational diffusion coefficient for increasing SUVs concentrations 9

Supp. Figure S7: Secondary structure propensities of $MapZ_{cyto}^{2TE}$ 10

Supp. Figure S8: Interaction of phosphomimetic $MapZ_{cyto}^{2TE}$ and wild-type $MapZ_{cyto}^{WT}$ with monomeric FtsZ, polymeric FtsZ, and SUV 11

Supplementary References **12**

Supplementary material and methods

Relaxation data acquisition and processing. Relaxation studies were performed on a 125-μM ^{13}C,^{15}N-uniformly labeled $MapZ_{cyto}$ sample in 30 mM HEPES buffer at pH 7.5 containing 50 mM KCl. ^{15}N-longitudinal relaxation, ^{15}N-transverse relaxation and ^{1}H-^{15}N heteronuclear NOE experiments were recorded at 5 °C on a Bruker AVANCE 14.1-T spectrometer equipped with a ^{1}H,^{13}C,^{15}N-cryoprobe using spin-relaxation pulse sequences from the NMRlib[1] library developed in IBS and derived from Farrow et al., 1994.[2] Relaxation delays of 0.020, 0.120, 0.220, 0.320, 0.420, 0.620,0.820, 1.020, 1.220, 1.520 s, and 8.48, 25.44, 42.40 (× 2), 59.36, 76.32, 110.24, 144.16, 178.08, 245.92 ms were used to determine longitudinal and transverse relaxation rate constants, respectively. R_1 and R_2 rates were fitted from the evolution of the resonance intensity as a function of the relaxation delay by the dedicated module in CcpNmr Analysis 2.4.1.[3] Standard deviations on these values were calculated from Monte Carlo simulations during the fitting procedures. $\{^{1}H\}^{15}N$-NOE values were determined by the comparison of the intensities of each amide resonance with and without a 3-s saturation period. Standard deviations were calculated from errors on peak intensities.

DOSY data acquisition and processing. 1D ^{13}C-filtered ^{1}H Diffusion Ordered Spectroscopy (DOSY) experiments[4] were recorded on a 105-μM and 100-μM ^{15}N-uniformly labeled $MapZ_{cyto}$ and $MapZ_{cyto}^{2TE}$ sample, respectively, in 50 mM Tris buffer at pH 7.5 containing 150 mM NaCl. Data were collected on an Avance III Bruker spectrometer operating at 950 MHz ^{1}H frequency at 5°C. ^{13}C pulses were EBURP 90° and REBURP 180° shaped pulses centered in the methyl region with a 3 ppm excitation window. A gradient diffusion time of 0.4 s was used and a total of 32 scans were collected for each 1D spectrum. Spectra were collected for variable gradient field strengths with the following typical values of 1.016, 4.269, 7.522, 10.775, 14.028, 17.280, 20.533, 23.786, 27.039, 30.292, 33.544, 36.797, 40.050, 43.303, 46.556, and 49.808 G cm^{-1}. Signal between 0.4 and 0.5 ppm was integrated and intensities were fitted using in house Python script as a function of the gradient field strength with the following equation:

$$I = I_0 e^{-D(\gamma_H G \delta)^2\left(\Delta - \frac{\delta}{3} - \frac{\tau}{2}\right)}$$

where γ_H is the proton gyromagnetic ratio in rad s^{-1} G^{-1}, G is the gradient field strength in G cm^{-1}, Δ, δ, and τ are delays in s. Fits allowed to determine translational diffusion coefficients D of 4.785×10^{7} and 4.785×10^{7} cm^2 s^{-1} for $MapZ_{cyto}$ and $MapZ_{cyto}^{2TE}$, respectively, in these conditions. The Bruker pulse sequence and fitting scripts are fully available from the NMRlib package.[1]

These experiments were reproduced after successive additions of a 54 mM solution of 50-nm SUVs made POPG:CL in a 1:1 molar ratio for lipid to MapZ molar ratios varying between 0.13:1 to 62:1. The evolution of the diffusion coefficient as function of this ratio is detailed in the case of $MapZ_{cyto}$ in Supp. Fig. S6.

Supplementary Figures

Supplementary Figure S1. Multiple sequence alignment of the cytoplasmic domain of MapZ used to calculate conservation scores with Consurf. Sequence alignment was built within Consurf (http://consurf.tau.ac.il/) from the 159 N-terminal residues of *S. pneumoniae* MapZ using built-in MAFFT algorithm. Homologs were collected from UniRef90 with BLAST as a search algorithm. Conservation scores were calculated with the Bayesian method. All protein sequences come from MapZ proteins from *Streptococcaceae*. Conservation scores go from low values (1) in blue to high values (9) in red. Scores for residues with a yellow 1-letter code are considered as untrusted due to the insufficient number of sequences in which they are present.

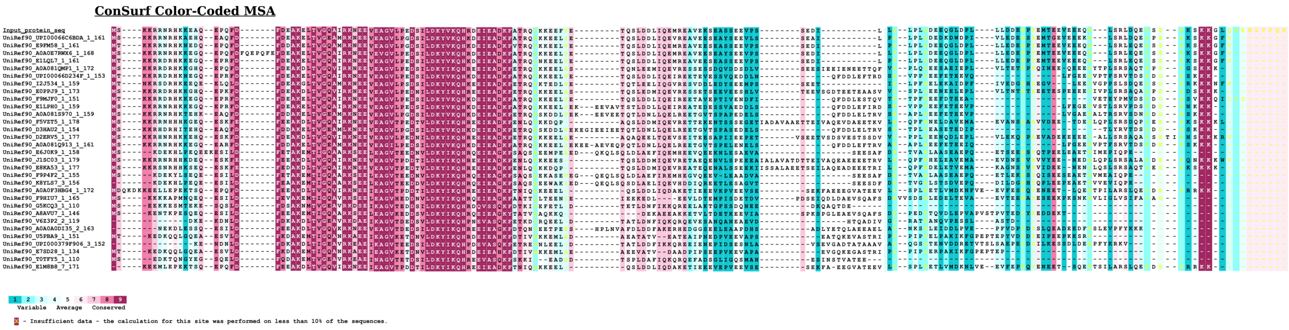

Supplementary Figure S2. **Relaxation data for backbone amide ^{15}N-resonances of MapZ$_{cyto}$.** From top to bottom: ^{15}N-longitudinal relaxation rate constants (R_1), ^{15}N-transverse relaxation rate constants (R_2) and ^{1}H-^{15}N heteronuclear nOe (HETNOE) values. Relaxation experiments were recorded on a Bruker AVANCE 14.1-T spectrometer at 5 °C on a 125-µM [^{13}C, ^{15}N]-uniformly labelled MapZ$_{cyto}$ sample in 30 mM HEPES buffer at pH 7.5 containing 50 mM KCl using pulse sequences from NMRLib.[1] Relaxation parameters were extracted as described in Supplementary materials and are shown as red histograms as a function of protein residue numbers. Black histograms represent error bars. Histogram values are not reported for residues yielding severely overlapped resonances.

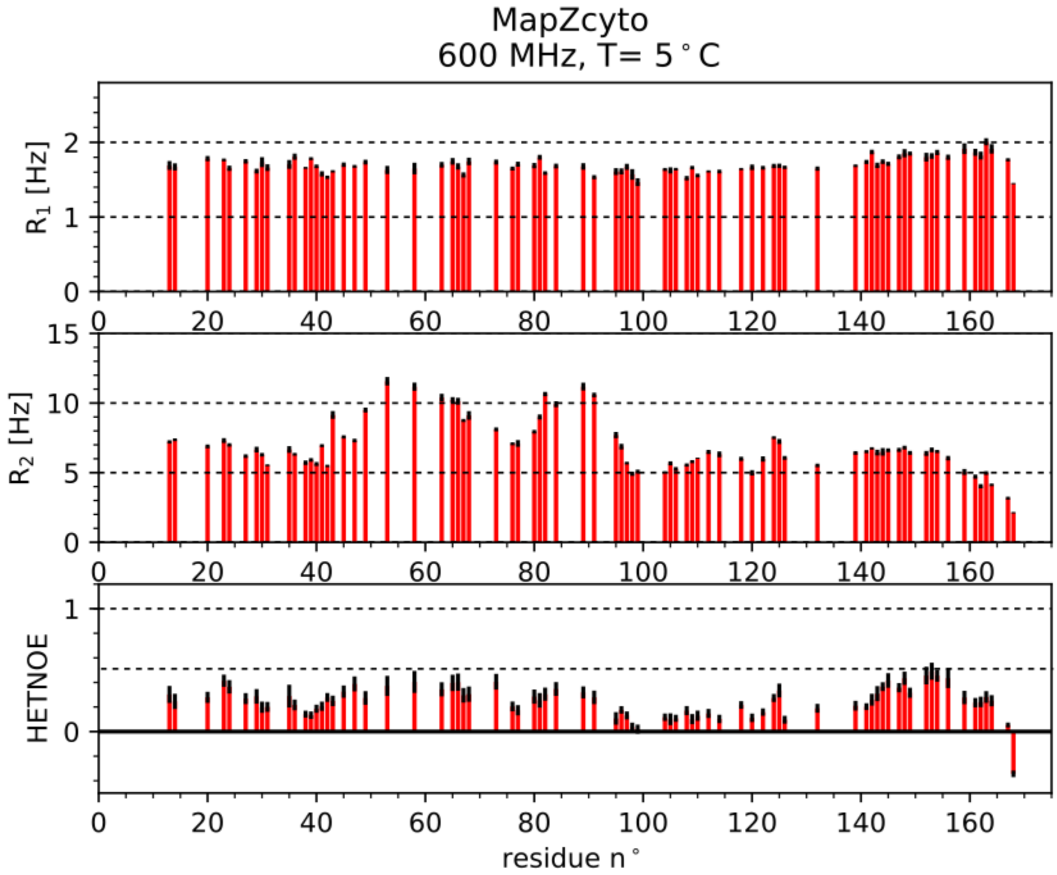

Supplementary Figure S3: **Control of FtsZ structure by NMR and polymerization by electron microscopy.** (a) 1D ^{1}H NMR spectra of the 110-µM non-labelled $FtsZ_b$ proteins in 30 mM HEPES, 200 mM KCl buffer at pH 7.5. Weak and broad signals in the methyl and amide regions in the [0.5, -0.5] and [8.5,9.5] ppm range, respectively, are used to verify that FtsZ is in a folded state. These 1D-sculpting spectra were acquired at 20 T on a Bruker Avance III spectrometer operating at 850 MHz proton frequency and 25 °C. (b) Negative-staining electron microscopy images were collected on $FtsZ_b$ in the absence (left) or in the presence (right) of 5 mM GTP after incubation with the protein at room temperature for 5 minutes. Long filaments observed in the right image imply that $FtsZ_b$ was properly folded and active to form FtsZ bundles. Both samples contained 5 µM $FtsZ_b$ in 30 mM HEPES, 200 mM KCl buffer at pH 7.5 and 5 mM $MgCl_2$. Data collected on $FtsZ_a$ have the same characteristics (data not shown).

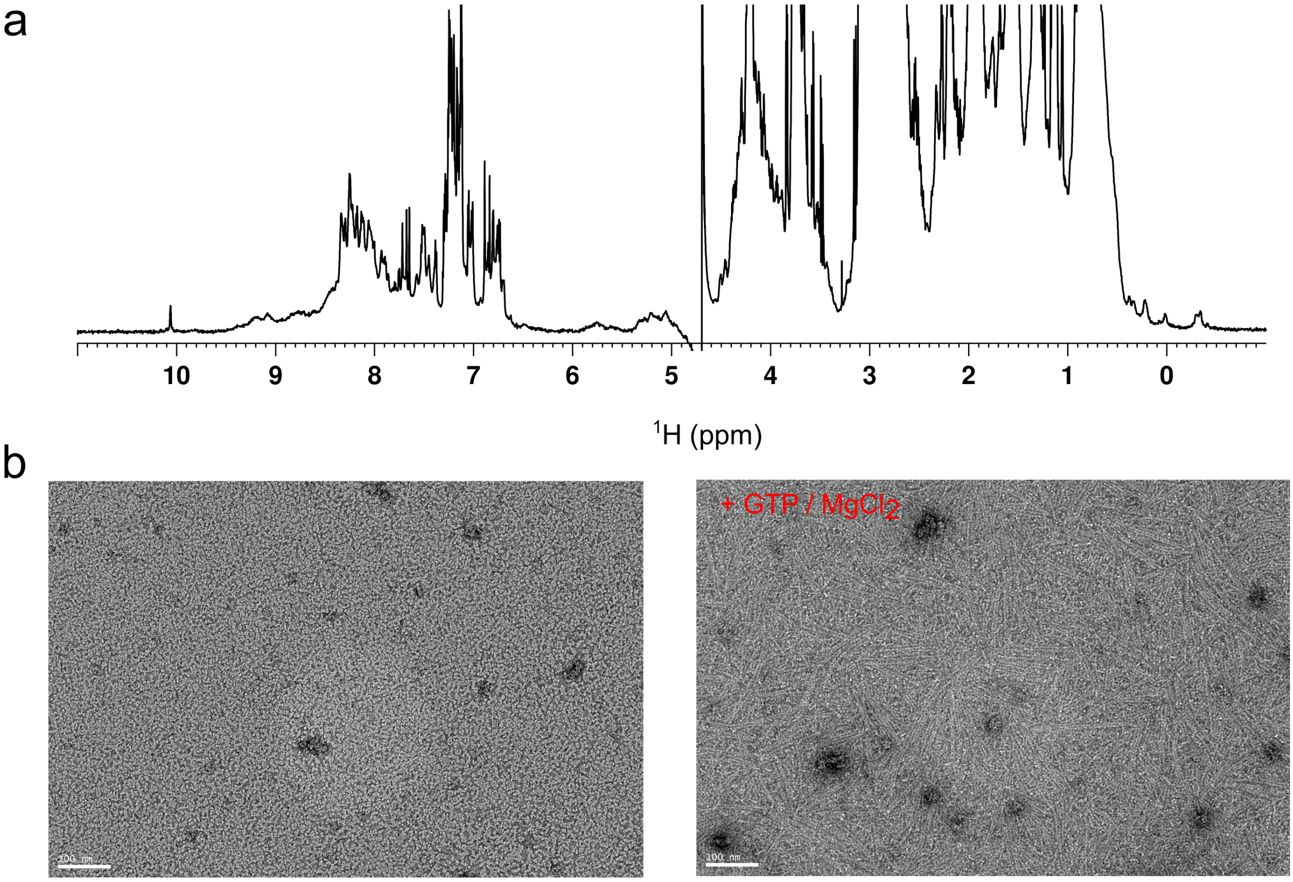

Supplementary Figure S4: **Interaction of $MapZ_{cyto}$ with monomeric and polymeric $FtsZ_b$.** Graphs showing the decrease of $MapZ_{cyto}$ resonance intensities measured in the ^{1}H–^{15}N BEST-TROSY spectra along the protein sequence at different $MapZ_{cyto}$:$FtsZ_b$ molar ratios. The series of samples at different $MapZ_{cyto}^{WT}$:$FtsZ_b$ molar ratios were prepared from 0.5 mM and 210 µM stock solutions of $MapZ_{cyto}$ and monomeric $FtsZ_b$ in 30 mM HEPES, 200 mM KCl buffer at pH 7.5. Final $MapZ_{cyto}$ concentration was 30 µM in each sample. In order to determine the impact of the oligomeric state of FtsZ on the interaction, experiments were initially done with a monomeric $FtsZ_b$ (in a). Subsequently 10 mM GTP and 10 mM $MgCl_2$ were added to the samples to polymerize $FtsZ_b$ (in b). NMR experiments were recorded at 16.4-T Bruker AVANCE spectrometer at 5 °C.

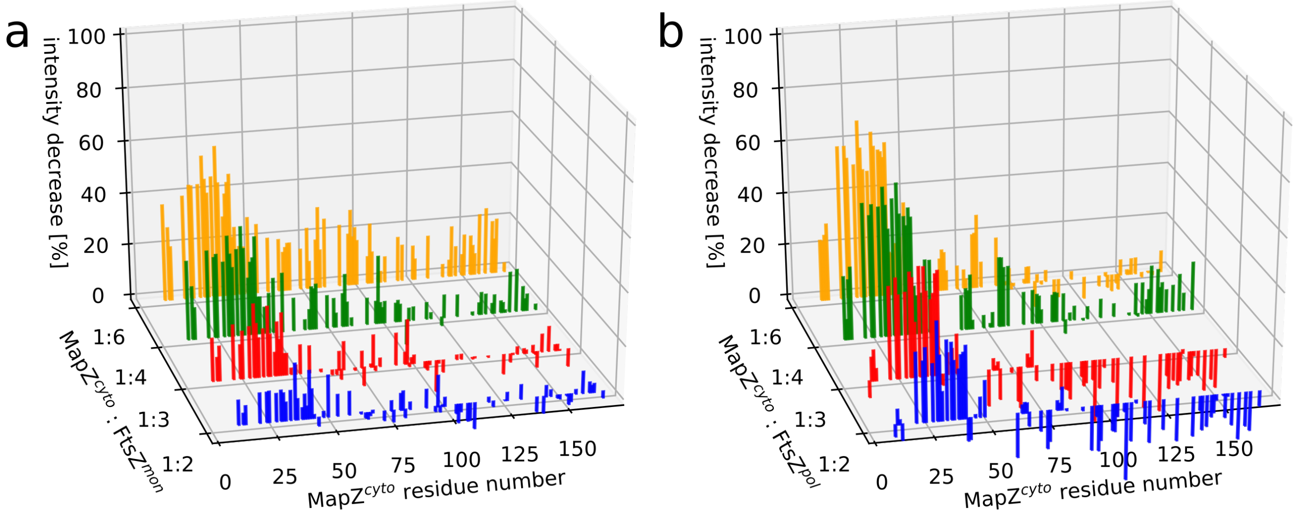

Supplementary Figure S5: Effect of MapZ$_{cyto}$ on FtsZ bundling. Negative-stain electron microscopy images of FtsZ filament bundles imaged with a calibrated nominal magnification of 440 (a) or 23,000 (b). Images shown in panel (c) are magnifications of panel (b) regions containing isolated FtsZ filaments. FtsZ polymerization and filament bundling were performed for 15 min at room temperature in a buffer containing 50 mM HEPES at pH 7.6, 200 mM KCl, 5 mM $MgCl_2$, 5 mM GTP, 8% (wt/vol) PVA, in the absence (left panel) or in the presence of 50 µM MapZ$_{cyto}$ (middle panel) or 50 µM BSA (right panel).

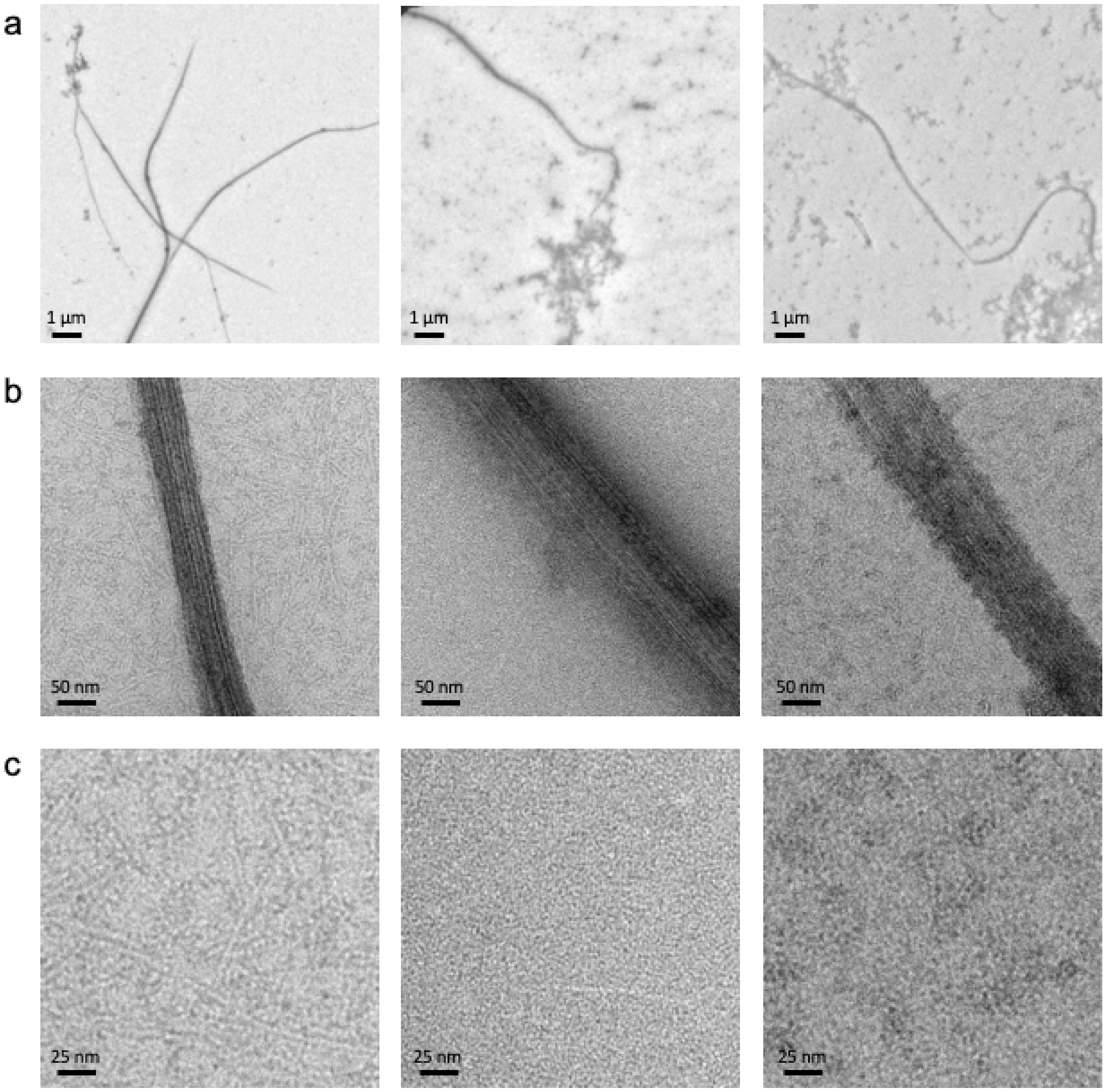

Supplementary Figure S6: **Evolution of the translational diffusion coefficient for increasing SUVs concentrations.** Translational diffusion coefficients D were determined using the ^{13}C-filtered ^{1}H DOSY experiment on a 105-µM ^{15}N- labeled $MapZ_{cyto}$ sample, in 50 mM Tris buffer at pH 7.5 containing 150 mM NaCl at 5°C for different MapZ to lipid molar ratios. The diffusion coefficient decreases when the lipid concentration is raised suggesting an interaction between the cytoplasmic domain of MapZ and the 1:1 POPG:CL 50-nm liposomes.

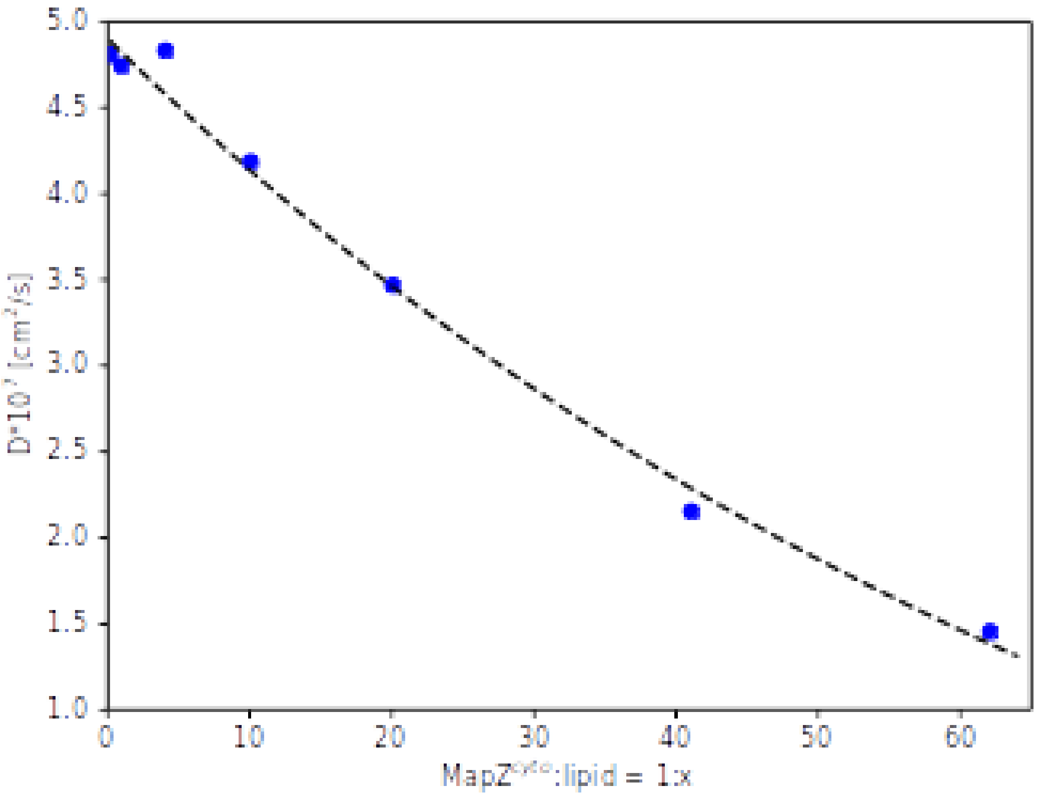

Supplementary Figure S7: Secondary structure propensities of MapZ$_{cyto}^{2TE}$. Neighbor corrected structural propensity (ncSP) scores calculated from C', Cα, and Cβ NMR chemical shifts of MapZ$_{cyto}^{2TE}$. ncSP scores reveal the propensity to form secondary structure (zero for random coil, positive for alpha-helices and negative for beta-sheets). Regions encompassing residues 49 to 68, 80 to 94, and 143 to 155 clearly show an alpha helical propensity as in the wild-type protein.

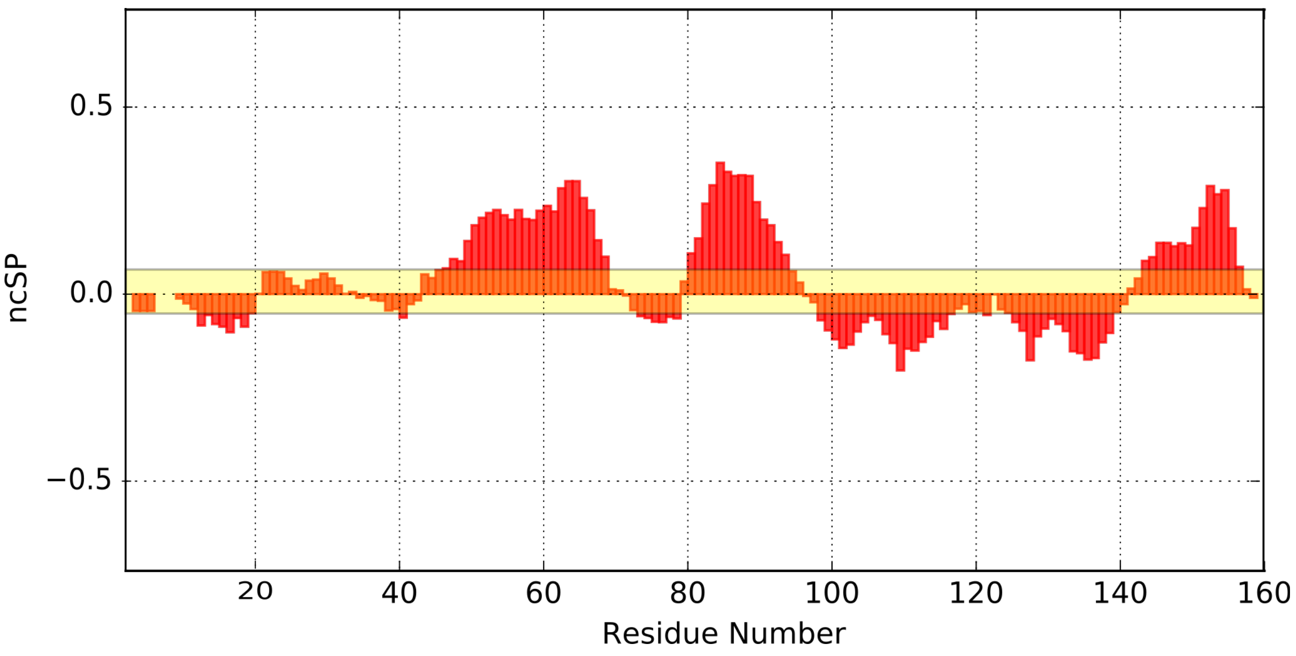

Supplementary Figure S8: Interaction of phosphomimetic MapZ$_{cyto}^{2TE}$ and wild-type MapZ$_{cyto}$ with monomeric FtsZ, polymerized FtsZ, and SUV. a-c) Graphs showing the decrease in the ^{1}H–^{15}N resonance intensity in BEST-TROSY spectra for each of the MapZ$_{cyto}$ amide resonances for different MapZ$_{cyto}^{2TE}$:FtsZ molar ratios. Interaction between (a) MapZ$_{cyto}^{2TE}$ and monomeric FtsZ, (b) MapZ$_{cyto}^{2TE}$ and polymerized FtsZ, and (c) MapZ$_{cyto}$ and monomeric FtsZ. Samples were prepared from low concentrated stocks of the proteins that were mixed together and concentrated. Final concentration of the MapZ$_{cyto}^{2TE}$ and MapZ$_{cyto}$ was 100 µM. Polymerization of FtsZ was initiated by addition of 10 mM GTP and 10 mM $MgCl_2$ to the MapZ$_{cyto}^{2TE}$:FtsZ samples. NMR experiments were recorded at 5 °C on a 14.1-T Bruker AVANCE spectrometer in the case of MapZ$_{cyto}^{2TE}$, and on a 16.5-T Bruker AVANCE spectrometer in the case of MapZ$_{cyto}^{WT}$. d-e) Graphs showing the decrease in the ^{1}H–^{15}N resonance intensity in BEST-TROSY spectra for each of the MapZ$_{cyto}$ amide resonances for different MapZ$_{cyto}$:lipid molar ratios. Interaction between (d) MapZ$_{cyto}$ and POPG:CL 1:1 50-nm SUVs, and (e) MapZ$_{cyto}^{2TE}$ and POPG:CL 1:1 50-nm SUVs. Initial concentration of the MapZ$_{cyto}^{2TE}$ and MapZ$_{cyto}$ samples was 100 µM. During experiments MapZ$_{cyto}$ concentration decreased slightly as small amounts of POPG:CL 1:1 50-nm SUVs stock solutions (with a 54 mM concentration in lipid) were gradually added to the samples. NMR experiments were recorded at 5 °C on a 16.5-T Bruker AVANCE spectrometer in the case of MapZ$_{cyto}^{2TE}$, and a 22.4-T spectrometer in the case of MapZ$_{cyto}$. All samples were prepared in 50 mM Tris, 100 mM NaCl buffer at pH 7.5. Resonance intensity decrease values were calculated as detailed in the Methods section of the main text.

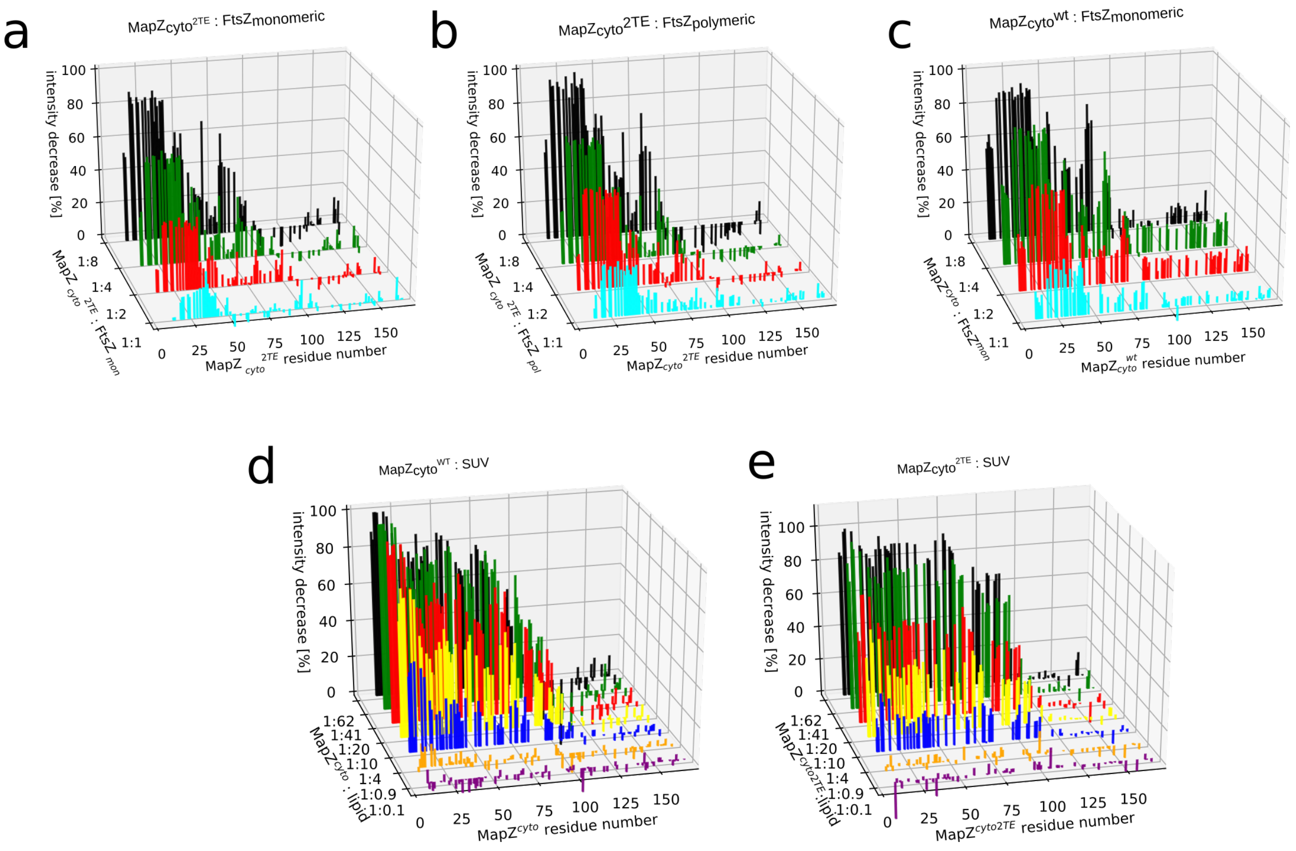

Supplementary References

1. Favier, A. & Brutscher, B. NMRlib: user-friendly pulse sequence tools for Bruker NMR spectrometers. *J. Biomol. NMR* (2019). doi:10.1007/s10858-019-00249-1
2. Farrow, N. a *et al.* Backbone dynamics of a free and phosphopeptide-complexed Src homology 2 domain studied by ^{15}N NMR relaxation. *Biochemistry* **33**, 5984–6003 (1994).
3. Vranken, W. F. *et al.* The CCPN data model for NMR spectroscopy: Development of a software pipeline. *Proteins Struct. Funct. Genet.* **59**, 687–696 (2005).
4. Johnson, C.S. Diffusion ordered nuclear magnetic resonance spectroscopy: Principles and applications. *Progress in Nuclear Magn. Res.* **34**, 203–256 (1999).
